# SNPs in inflammatory genes *CCL11*, *CCL4* and *MEFV* in a fibromyalgia family study

**DOI:** 10.1371/journal.pone.0198625

**Published:** 2018-06-21

**Authors:** Zhifang Zhang, Jinong Feng, Allen Mao, Keith Le, Deirdre La Placa, Xiwei Wu, Jeffrey Longmate, Claudia Marek, R. Paul St. Amand, Susan L. Neuhausen, John E. Shively

**Affiliations:** 1 Department of Molecular Imaging and Therapy, Beckman Research Institute, City of Hope, Duarte, CA, United States of America; 2 Department of Molecular Medicine, Beckman Research Institute, City of Hope, Duarte, CA, United States of America; 3 Department of Biostatistics, Beckman Research Institute, City of Hope, Duarte, CA, United States of America; 4 R.P. St. Amand MD Inc, Marina Del Rey, CA, United States of America; 5 Department of Population Sciences, Beckman Research Institute, City of Hope, Duarte, CA, United States of America; University of Texas Rio Grande Valley, UNITED STATES

## Abstract

**Background:**

Fibromyalgia (FM) is a chronic pain syndrome with a high incidence in females that may involve activation of the immune system. We performed exome sequencing on chemokine genes in a region of chromosome 17 identified in a genome-wide family association study.

**Methods and findings:**

Exome sequence analysis of 100 FM probands was performed at 17p13.3-q25 followed by functional analysis of SNPs found in the chemokine gene locus. Missense SNPs (413) in 17p13.3-q25 were observed in at least 10 probands. SNPs rs1129844 in *CCL11* and rs1719152 in *CCL4* were associated with elevated plasma chemokine levels in FM. In a transmission disequilibrium test (TDT), rs1129844 was unequally transmitted from parents to their affected children (p< 0.0074), while the *CCL4* SNP was not. The amino acid change (Ala23Thr), resulting from rs1129844 in *CCL11*, predicted to alter processing of the signal peptide, led to reduced expression of CCL11. The variant protein from *CCL4* rs1719152 exhibited protein aggregation and a potent down-regulation of its cognate receptor CCR5, a receptor associated with hypotensive effects. Treatment of skeletal muscle cells with CCL11 produced high levels of CCL4 suggesting CCL11 regulates CCL4 in muscle. The immune association of FM with SNPs in *MEFV*, a chromosome 16 gene associated with recurrent fevers, had a p< 0.008 TDT for a combined 220 trios.

**Conclusions:**

SNPs with significant TDTs were found in 36% of the cohort for *CCL11* and 12% for *MEFV*, along with a protein variant in CCL4 (41%) that affects CCR5 down-regulation, supporting an immune involvement for FM.

## Introduction

Fibromyalgia (FM) is a pain-associated syndrome affecting the musculoskeletal system in 2–5% of the general population [1, 2]. Besides wide-spread pain in all four quadrants of the body, the majority of patients suffer from sleep disorder, mental fog, and irritable bowel syndrome [3, 4]. Family studies have established a genetic basis for the syndrome [5], including a genome-wide association (GWAS) study [6] that identified a region of chromosome 17 (17p11.2–q11.2) containing a chemokine-gene-cluster locus. A recent study examined gene expression profiles in peripheral blood defining a ten-gene signature of which several fit an inflammatory pathway [7]. While several single-nucleotide polymorphisms (SNPs) in pain-related genes were examined as the presumptive cause of FM [7–11], we [12, 13] and others [14], have proposed that the preponderance of the syndrome in females, as high as 85% [1], and the association of FM with symptoms such as muscle aches, sleep disorder and irritable bowel, suggests the syndrome may have an immune component. In a previous study [12], we showed that several inflammatory chemokines were elevated in FM, including CCL11 and CCL4, genes located in a chemokine gene cluster on chromosome 17. This chemokine locus is associated with atopic dermatitis [15] and IBD [16], both immune-related disorders. As a follow-up to the GWAS FM family study on chromosome 17 [6], we performed functional studies on the chemokine gene-cluster region on chromosome 17 by exome sequence and functional analyses.

Since chronic elevation of these chemokines may exhibit a fever-like state with FM symptomology (muscle pain, etc.), we also analyzed SNPs on *MEFV*, a gene in which SNPs are associated with recurrent fevers and the activation of IL-1**β** in the inflammasome [17]. Since we had previously shown that 15% of FM patients in a cohort of 100 trios had a significant association with SNPs in *MEFV* [13], our new cohort of 120 trios allowed us to validate the results of the previous study and to perform analysis on the combined cohorts of 220 trios. The results show that in 12% of the combined cohorts, *MEFV* SNPs are associated with FM. In a separate study of 187 Turkish patients, Karakus also found a significant association of FM with *MEFV* SNPs [18]. Given that 12% of our cohort had an association with SNPs in MEFV and 36.8% with SNPs in CCL11, our results suggest that polymorphisms of the immune system may play a causative role in a substantial number of FM patients.

## Materials and methods

### Materials

Recombinant human CCL3 (MIP-1**α**), CCL4 (MIP-1**β**), CCL11 (Eotaxin), CCL1 (MCP-1), IL-13, GM-CSF, IL-4, and IFN**γ** were obtained from ProSpec Protein-Specialists (East Brunswick, NJ, USA); LPS from E. coli (055:B5) were purchased from Sigma-Aldrich (St. Louis, MO, USA); recombinant human CCL4 variant (S80T) was from Kingfisher Biotech, Inc (St Paul, MN, USA); anti-human CCR5-PE (CD195, Clone# J418F1), anti-human CCR1-APC (CD191, Clone# 5F10B29), anti-human CD4-PerCP (Clone# OKT4) and anti-human CD3-FITC (clone# SK7) were from Biolegend (San Diego, CA, USA); Indo-1-AM was from Molecular Probes (Eugene, OR).

### Participants

This study was approved by the Institutional Review Board of City of Hope National Medical Center (IRB 04186). A total of 220 patients with fibromyalgia and their parents were recruited into the study. Written informed consents were obtained from participants, or in the case of minors, their parents. Patients were diagnosed using American College of Rheumatology criteria [19] including musculoskeletal pain that exists for over three months, associated with fatigue, depression, cognitive difficulty and irritable bowel. Patients with rheumatoid arthritis and systemic lupus erythematosus were excluded from the study; clinical characteristics of the patient population have been previously described [13]. Unrelated, gender and age- matched control subjects were used for comparison of cytokine/chemokine levels.

### Exome sequencing

Genomic DNA from peripheral blood lymphocytes or saliva was sonicated to produce fragment sizes of 200–250 bp and Illumina adapters were ligated to generate the libraries. Each library was hybridized to biotinylated cRNA oligonucleotide baits from the SureSelect Human All Exon kit (Agilent Technologies), purified by streptavidin-bound magnetic beads, and amplified for 12 cycles. Paired-end (80 × 80 bp) sequencing was conducted using an Illumina Genome Analyzer IIx or Hiseq 2000 (Illumina Inc.). The exome probes covered 50 Mb of the human genome, corresponding to the exons and flanking intronic regions of all genes in the National Center for Biotechnology Information Consensus CDS database (accession no. SRA072282).

### PCR and amplicon sequencing

DNA was isolated from peripheral leukocytes or saliva, using the QIAamp DNA Blood Mini Kit (Qiagen) and Oragene DNA Self Collection Kits (DNA Genotek) according to the manufacturers' instructions. The coding exons with known SNPs from deep sequencing were amplified by PCR in a total volume of 20 µl with 10 mM Tris-HCl, pH 8.3, 50 mM KCl, 1.5 mM MgCl2, 200 µM of dNTPs, and 0.2 µM of primers. 1 U of Ampli-Taq Gold (Roche) and 20 ng of genomic DNA were added. PCR reactions were performed on the GeneAmp PCR System 9700 (Applied Biosystems) with denaturation at 94°C for 10 min, and then denaturation at 94°C for 15 sec, annealing at 60°C for 30 sec, and elongation at 72°C for 1 min for a total of 35 cycles and a final elongation for 10 min at 72°C. The amplicons were purified by ExoSAP-IT and sequenced with the ABI PRISM 3730 (Applied Biosystems). The sequences of PCR primers are available upon request. Genomic and amino acid sequences for candidate genes were collected from Ensemble.

### Transient expression of WT and varCCL11

Expi 293F™ cells, ExpiFectamine 293 transfection kit, Expi 293 expression medium, and Opti-MEM reduced serum medium were purchased from Life Technologies Corporation (Carlsbad, CA). The expression vector containing WT CCL11 human cDNA ORF clone and anti-DDk monoclonal antibody (Clone 4C5) were purchased from OriGene Technologies, Inc (Rockville, MD). VarCCL11 (Ala23Thr) was generated using QuikChange Lightning Multi Site-Directed Mutagenesis Kit (Agilent Technologies, Carpinteria, CA) and confirmed by sequence analysis. Expi 293 transfections were performed according to the manufacturer's protocol. Briefly, ExpiFectamine 293 transfection reagent and plasmid DNA were separately diluted in OptiMEM complexation medium. Following a 5-minute incubation, ExpiFectamine 293 and DNA mixtures were combined and incubated for an additional 20 min. The ExpiFectamine 293-DNA-OptiMEM mixture was then added to cells. Enhancer 1 and Enhancer 2 were added to transfected cultures 16 hours after transfection. Supernatants were harvested from transient transfected Expi 293F cell culture at day 7, sterile filtered and analyzed by SDS gel electrophoresis on a 12% polyacrylamide gel and visualized with Coomassie brilliant blue. CCL11 concentration in the supernatant was measured using human CCL11 ELISA kit (Aviva Systems Biology Corp).

### Functional studies

Chemokine and cytokine levels were compared in unrelated healthy controls and FM cases with wild type or variant alleles using the Luminex 100/200 system. Peripheral blood mononuclear cells (PBMCs) were isolated from citrated blood of healthy donors by Ficoll-Hypaque (Pharmacia Biotech, Uppsala, Sweden) density gradient centrifugation as recommended by the manufacturer. Monocytes were collected and separated from PBMCs using EasySep™ Human Monocyte Enrichment Kit (StemCell Technologies, Vancouver, BC, Canada). The purity of monocytes were stained with anti-CD14-FITC and checked by using flow cytometry FACSCanton II (BD Biosciences, San Jose, CA). Cells were suspended in RPMI 1640 supplemented with 100 U/mL penicillin, 100 mg/mL streptomycin, 1 mM L-glutamine and 10% FBS with cells concentration 1 × 10^6^/mL. For monocyte-derived immature dendritic cell differentiation, monocytes were treated with 1,000 U/mL GM-CSF and 500 units/mL IL-4 for two days. The human CEM.NKR CCR5 T-cell line were kindly provided from Dr. John Burnett’s lab at City of Hope and grown in RPMI1640 with 10% FBS, 2 mM L-glutamine and antibiotics at 5% CO2, 37°C. Myoblast and skeletal muscle cell medium BulletKit® (containing skeletal muscle cell basal medium, EGF, insulin, dexamethasone, Gentamicin sulfate, Amphotericin-B, and Bovine serum albumin) were purchased from Cambrex Bio Science Walkersville, Inc. Cells were seeded in 24-well plates at 10,000 cells/cm^2^, grown to confluency, treated with LPS, MCP-1, Eotaxin, IL-13, or IFN**γ** for 48 h, and supernatants were harvested for cytokine multiplex analysis. One-way MLR was carried out by co-culturing 1 × 10^6^/ mL PBL (responder cells) and 1 × 10^6^/ mL irradiated allogeneic PBL (stimulator cells, 900 cGy) in the T-125-cm^2^ tissue culture flask for 9 days. T cell surface CCR5 expression was detected by using flow cytometry. Eosinophils were isolated using EasySep™ Human Eosinophil Enrichment Kit (StemCell Technologies, Vancouver, BC, Canada). The purity of eosinophils was >90% assessed by anti-human CCR3-APC antibody and anti-human CD16-PerCP antibody (Biolegend, San Diego). Eosinophils were treated with commercial CCL11 (Biolegend, San Diego), expressed WT CCL11, or varCCL11 for 2 hours and CCR3 down-regulation was analyzed using anti-human CCR3 antibody, Canton II Flow cytometry and Flowjo software.

### Calcium mobilization assay

Immature DCs, CEM.NKR CCR5 T-cell line and 9-day one-way MLR cells at 10^7^/mL were loaded with 5µM Indo-1-AM for 7 min at 37°C and kept at room temperature for an additional 10 min. Cells were washed with a 5-fold excess of cold PBS and centrifuged at 200 × g for 10 min at 4°C. Flow cytometric measurements of intracellular Ca^2+^ was performed using a BD LSRFortessa™ (BD Biosciences, San Jose, CA, USA). Samples were excited at 351 nm and emission fluorescence was recorded at both 405/20 and 485/20 nm for Indo-1 AM. FACS analyses were performed on suspensions of 2 × l0^6^ cells/mL in PBS with calcium and magnesium. Base-line fluorescence was determined and cells were then stimulated with CCL4 (20 ng/mL), variant CCL4 (20 ng/mL) and ionomycin (1 µM) were added to suspended cells and monitored as described. The ratio of 405:485 nm was detected as intracellular calcium mobilization ([Ca2+]i).

### Mass spectrometry and size exclusion chromatography (SEC)

The molecular sizes of CCL11, varCCL11, CCL4 and variant CCL4 were determined by electrospray ionization mass spectrometry on an Obitrap Fusion mass spectrometer (Thermo Scientific). Size exclusion chromatography was performed on a Superdex 200 10/30 column (GE Healthcare Life Sciences) run at 0.5 mL/min in PBS. UV detection was performed at 214 and 280nm on a GE Akta Purifier HPLC.

### Statistical analysis

Assay results were means ± standard deviation (SD) or ± standard error (SE). Student’s t tests were used for comparisons of chemokine levels (P values were unpaired, two-sided). Statistical significance in the text refers to p < 0.05 for cytokine data, and transmission disequilibrium tests (TDTs), and p < 0.0125 for the four new TDTs of chemokine genes. The exact version of the TDT for MEFV was previously described (17).

## Results

### Identification of a *CCL11* variant as a possible marker of FM

Since FM occurs in families, there are likely susceptibility genes. Although several studies based on candidate gene hypotheses have identified associated SNPs, their functional significance has not been studied. Recently, a GWAS study of 116 FM families identified association with a locus at chromosome 17p11.2–q11.2 [6]; however, no candidate gene(s) emerged from that study. As a first approach to identify the candidate gene(s), we conducted exome sequencing of an expanded region of chromosome 17 (17p13.3-q25) from 100 probands with FM, and identified 4332 SNPs that were also found in the ExAc data base. To reduce the number of SNPs for analysis, we selected only SNPs occurring in at least 10% of the 100 FM probands, twice the higher end of the estimated frequency of 2 to 5% of FM in the general population. Four hundred and thirteen SNPs met these criteria (**S1 Table**). Based on the hypothesis that FM has an immune component, we further selected only those SNPs found in the chromosome 17 cluster of 18 chemokine genes [20]. Based on these criteria, only four SNPs in four chemokine genes (CCL11, CCL8, CCL23 and CCL4) were further studied (**Table 1, upper panel)**. The allele frequencies of these four SNPs were similar to the ExAC dabase, with sequencing data from over 60,000 unrelated individuals [21] (**Table 1, middle panel**). An example of sequencing data for CCL11 is shown in **S1 Fig**. Since the incidence of FM is common in the general population and its occurrence is associated with an environmental trigger such as trauma [22] or a chronic inflammatory condition such as IBS [4], the similar allele frequency in the general population was not surprising. This is similar to the 1% incidence of celiac disease, where 95% express HLA-DQ2 that occurs in 25% of the Caucasian population [23], suggesting that a genotype alone is necessary but not sufficient for development of a disease.

**Table 1:** SNPs of immune related genes in chromosome 17 from FM cohorts and transmission analysis from FM trios^1^^,^^2^^,^^3^.

			FM Patients					
**Gene**	**AA Change**	**AA Pos**	**Ref Allele**	**Hetero**	**Homo**	**Var Allele**	**MAF**	**ExAc Freq**
*CCL11*	Ala>Thr	23	65	29	6	41	20.50%	18.32%
*CCL8*	Lys>Gln	69	76	22	2	26	13.00%	15.51%
***CCL23***	**Val>Met**	123	4	26	70	166	**83.00%**	**81.03%**
*CCL4*	Ser>Thr	80	53	41	6	53	26.50%	23.22%
								
			**Ref Allele**	**Hetero**	**Homo**	**Var allele**	**MAF**	**ExAC Freq**
*CCL11*	Ala>Thr	23	139	72	9	90	20.45%	18.32%
*CCL8*	Lys>Gln	69	86	30	4	38	15.83%	15.31%
*CCL23*	**Met>Val **	123	80	33	7	47	**19.58%**	**18.97%**
*CCL4*	Ser>Thr	80	129	88	9	105	24.09%	23.22%
								
			**Transmitted**		**Not Transmitted**		**P value**	
*CCL11*	Ala>Thr	23	81		52		0.0074	
*CCL8*	Lys>Gln	69	33		37		NS	
*CCL23*	Met>Val	123	42		36		NS	
*CCL4*	Ser>Thr	80	80		77		NS	

^1^ Upper: raw Illumina sequencing data of 100 FM patients taken from **S1 Table**. MAF = minor allele frequency. Note: for CCL23 in the ExAc dbase Val>Met is reported as the MAF (bold type), but should be Met>Val. This discrepancy is corrected in the validation section (Middle). NS, not significant.

^2^ Middle: validated data by direct sequencing of 220 FM samples for CCL11 and CCL4, and 120 FM samples for CCL8 and CCL23. The corrected minor allele for CCL11 (Val) and the MAF are shown in bold.

^3^ Lower: transmission analysis includes 220 trios for CCL11 and CCL4, and 120 trios for CCL8 and CCL23. P-values are one-sided on the presumption that the variant allele confers higher risk of FM.

Using the transmission disequilibrium test (TDT), we analyzed the transmission of the four SNPs from parents to probands. Of the four SNPs, only rs1129844 of *CCL11* was significant (p = 0.0074) (**Table 1, lower panel**). Out of 220 FM cases in our combined cohorts, 72 were heterozygous and 9 were homozygous for G>A (rs1129844), with a minor allele frequency (MAF) of 19.58% compared to the ExAC dbase reported frequency of 18.32%. Overall, 36.8% of the 220 patients had at least one copy of this SNP.

**Fig 1:**
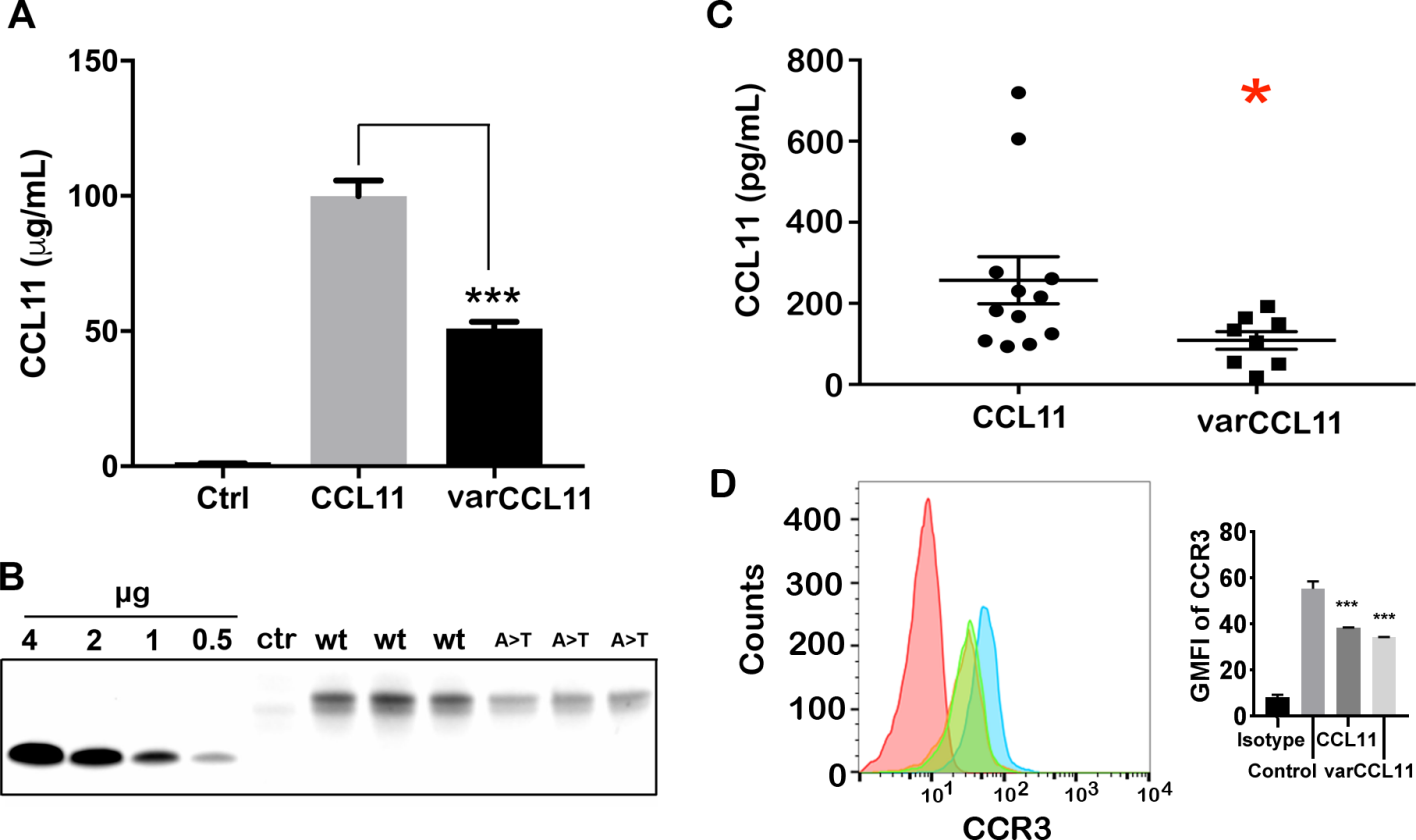
Expression of wild type and variant (Ala23Thr) CCL11. Plasmids encoding the genes for wild type and variant (Ala23Thr) CCL11 were expressed in HEK cells and the expression levels determined by (**A**) EIA (triplicates; ***, p< 0.001) and (**B**) Coomassie Blue staining (triplicates). Control media (**A**: Ctrl) had undetectable levels of CCL11. Recombinant CCL11 standards (**B**: left) were used to quantitate protein expression of wild type (WT) and variant (A>T) CCL11 (**B**: right). Control media (Ctr) had <0.05 µg expression of CCL11. The difference in migration of recombinant standards vs expressed protein on SDS gel electrophoresis is due to glycosylation (confirmed by mass spectrometry, data not shown). **C**: Control monocytes expressing either wild type (n = 12) or varCCL11 (heterozygous, n = 8) were incubated in media for 18 hrs and CCL11 levels were measured (*, p<0.026). **D**: Eosinophils isolated from a donor were stained for CCR3 expression before (blue) or after incubation with equal amounts of WT (green) or varCCL11 (orange). The green and orange plots are identical. Isotype control (red).

The predicted amino acid change (Ala23Thr) for *CCL11* rs1129844 is potentially important, since this is the exact position at which the signal peptidase cleaves pre-CCL11 to generate mature CCL11 during processing in the endoplasmic reticulum (ER). In other proteins where a similar mutational position is observed, the protein is often not expressed [24, 25]. To examine the effect of this SNP, we expressed plasmids containing wild type or varCCL11 in HEK cells and analyzed the supernatants for CCL11 expression (**Fig 1A and 1B**). We observed that the mutation affected both the intrinsic expression levels and activity of varCCL11. Analysis of the expressed product by mass spectrometry (data not shown) revealed altered processing to position +2 and confirmed the presence of O-glycosylation as previously shown by Noso et al. [26]. The supernatant levels of CCL11 in unstimulated monocytes from heterozygous controls were lower than that from homozygous WT controls (**Fig 1C**, p<0.026), suggesting that even one variant allele leads to lower CCL11 production. However, both WT and varCCL11 down regulate CCR3 on eosinophils (**Fig 1D**), demonstrating equivalent function in eosinophils, the major target of CCL11. Thus, the variant allele may function to limit the amount of CCL11 that can be produced.

Given the report that SNP rs111568837 in the promoter of *CCL11* correlated with the incidence of atopic dermatitis in an Italian cohort [15], we investigated whether rs111568837 also was associated with FM. However, the transmission of this SNP from parents to probands was not statistically significant **(S2 Table)**. Interestingly, the *CCL11* coding SNP rs1129844 that was significant in our cohort was not statistically significant in the Italian atopic dermatitis cohort.

**Fig 2:**
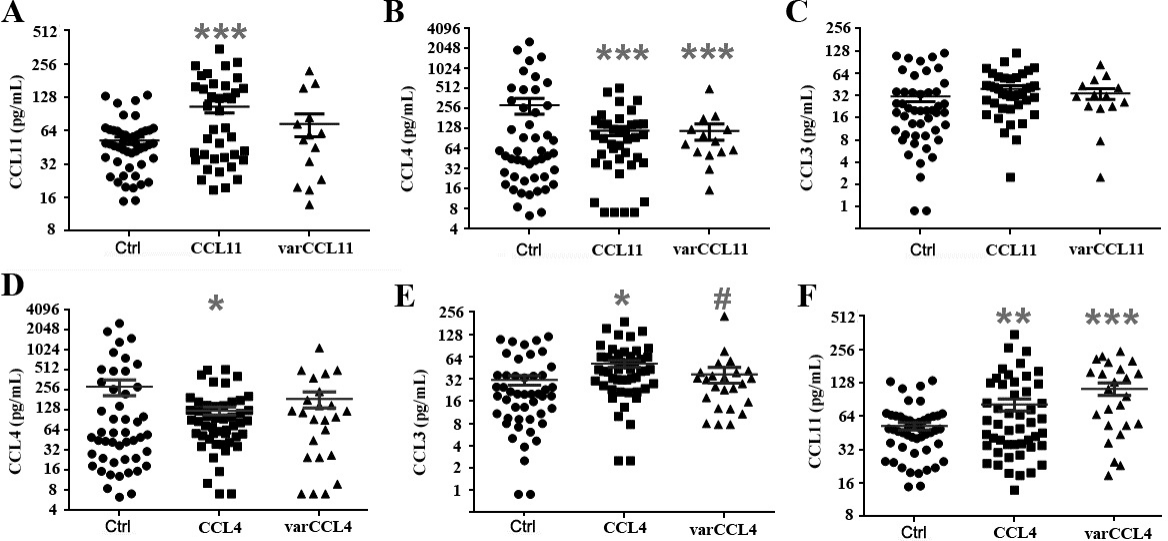
Plasma levels of CCL11, CCL4 and CCL3 in female FM patients with varCCL11 or varCCL4. **A-C**, CCL11, CCL4, and CCL3 levels in female healthy controls (Ctrl, n = 48) vs female FM patients with wild type CCL11 (n = 19) or var CCL11 (n = 14). **D-F**, CCL4, CCL3 and CCL11 levels in female healthy controls (Ctrl, n = 48) vs wild type CCL4 (n = 49) or varCCL4 (n = 24) in female FM patients. (* P<0.05, ** P<0.01, *** P<0.001 in comparison with female healthy controls; # P<0.05 in comparison with female FM patients with wild type CCL4.

Since we previously found significantly high expression of CCL11 in FM patients [12], we re-examined their expression levels based on their *CCL11* genotype (**Fig 2A**). As expected, probands with WT CCL11 had high average expression (p< 0.001 vs controls), while those with varCCL11 had a distribution not statistically distinguishable from WT probands or controls. Thus, CCL11 expression is associated with FM, resulting in high levels in those with wild type alleles, but less so in those with varCCL11, likely due to a functional defect in expression. The overall high expression of CCL11 in FM patients may be interpreted as causal or compensatory. Causal would predict that CCL11 associated SNPs would lead to higher levels of CCL11. However, given that the CCL11 SNP identified is associated with lower levels of CCL11, a compensatory mechanism is also possible, in that patients with this SNP would be predicted to not adequately raise their levels of CCL11 in response to an as yet unknown trigger of the syndrome. If low expression of CCL11 in patients with varCCL11 can be verified in a larger study and/or shown to be associated with eosinophils, the primary cellular target of CCL11, this would provide further evidence that CCL11 production is a compensatory rather than a casual mechanism in FM.

Since CCL4 expression is also high in FM patients, further analysis revealed that the levels of CCL4 but not the related chemokine CCL3 were decreased in those with varCCL11 compared to controls, suggesting CCL11 and CCL4 are both involved in FM (**Fig 2B and 2C**). These data suggest that CCL11 SNPs that lead to lower levels of CCL11 also affect the production of CCL4. If verified in larger studies, then CCL4 may also be produced as a compensatory mechanism in FM and depend on CCL11 production.

### Identification of a *CCL4* variant as a possible marker of fibromyalgia

Although the *CCL4* SNP examined in **Table 1**was not significant by TDT, given the report that the peripheral CCL4-CCR5 axis contributes to neuropathic pain [27] suggesting a possible connection to the widespread pain experienced in FM, we performed functional analyses on this SNP. In addition to widespread pain, many FM patients exhibit orthostatic hypotension as a symptom [28], another possible connection to the CCL4-CCR5 axis, since Maraviroc, a CCR5 antagonist for the treatment of HIV causes hypotension as a side effect [29]. We tested whether the conservative amino acid change (Ser80Thr) in the var*CCL4* could result in a functional change in the protein. Notably, closely related CCL3 and its variants differ in their dimer vs aggregation states [30] when they form homodimers with themselves or heterodimers with CCL4 [31]. To determine if a similar situation occurred with CCL4 or varCCL4, we determined their molecular sizes. The wild-type protein behaves as a relatively sharply migrating homodimer at a molecular size of 16 kDa (**S2A Fig)**, whereas the variant migrates as two broad peaks, one at high molecular weight (>300 kDa) and the other at 16 kDa (**S2B Fig**). Thus, there is evidence that the variant exists as a high molecular weight aggregate that slowly reverts to a dimer upon dilution. It is likely that the aggregation state of the variant occurs during the secretion process from cells where protein concentrations are high, and slowly reverts to dimers upon dilution into circulation.

Due to the possible aggregation effects for the *CCL4* variant, we analyzed the plasma levels of several cytokines and chemokines in FM cases with wild type and varCCL4 compared to healthy controls. VarCCL4 significantly affected the FM plasma levels of CCL4 compared to controls **(Fig 2D and 2E)**. In addition, CCL11 levels were higher in wild type CCL4 and even higher in varCCL4 compared to controls (**Fig 2F**). These results further suggest a possible relationship between the two chemokines and their variants.

**Fig 3:**
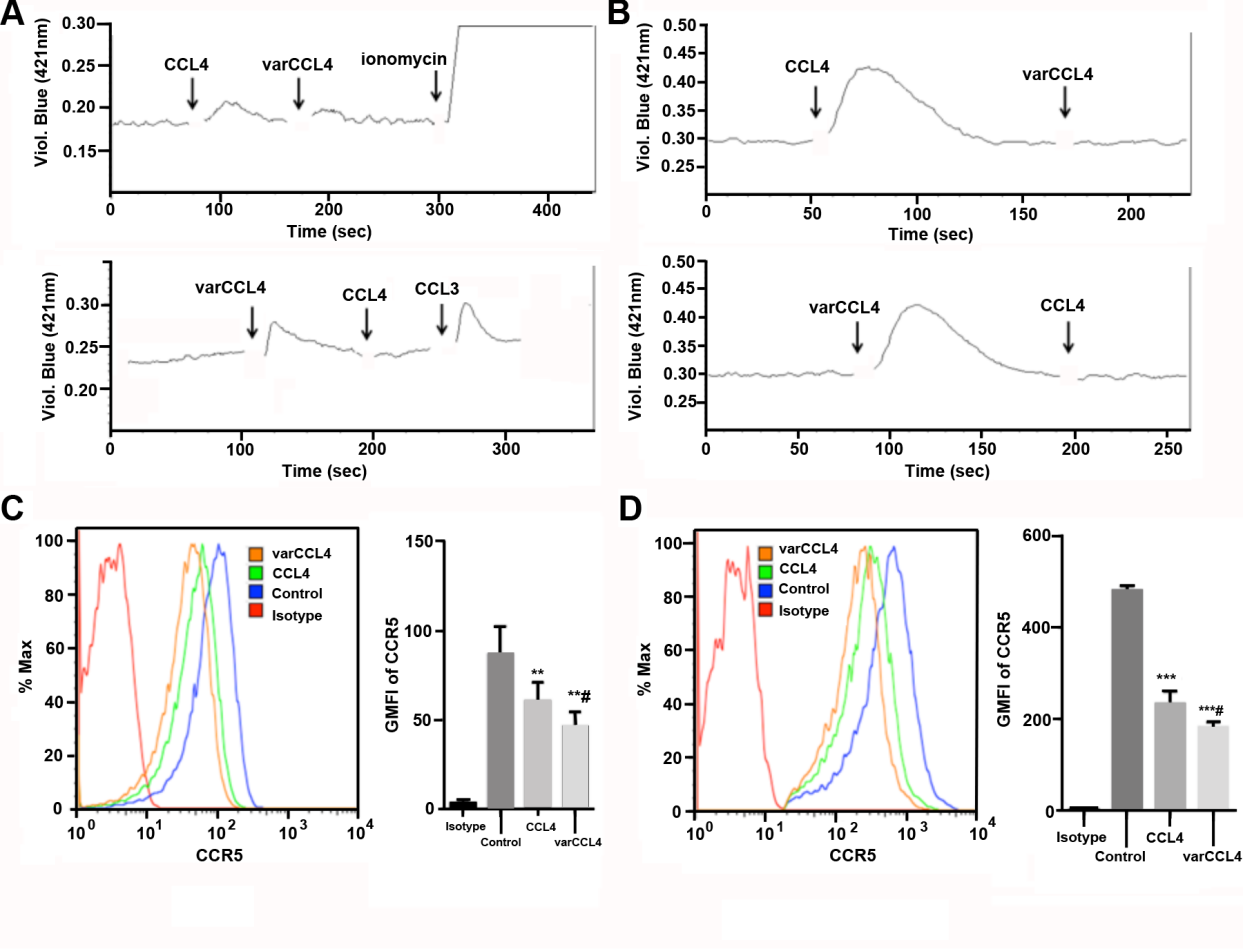
Functional analyses of WT vs varCCL4 in immature dendritic cells or CEM.NK^R^ CCR5 T-cells. **A-B**. WT CCL4 and varCCL4 both stimulate and cross-block calcium mobilization in CCR5 positive immature dendritic cell (**A**) or CEM.NK^R^ CCR5 T-cells (**B**). Ionomycin and CCL3 were used as positive controls. **C-D**. varCCL4 induces greater down-regulation of cell surface CCR5 than WT CCL4 on immature dendritic cells (**C**) and CEM.NK^R^ CCR5 T-cell line (**D**). Cells were treated with 80 ng/mL of each CCL4 species for 3 hours, surface CCR5 was stained with anti-CCR5 antibody and analyzed by flow cytometry (** P<0.01, *** P<0.001 in comparison with untreated control; # P<0.05 in comparison with WT CCL4).

**Fig 4:**
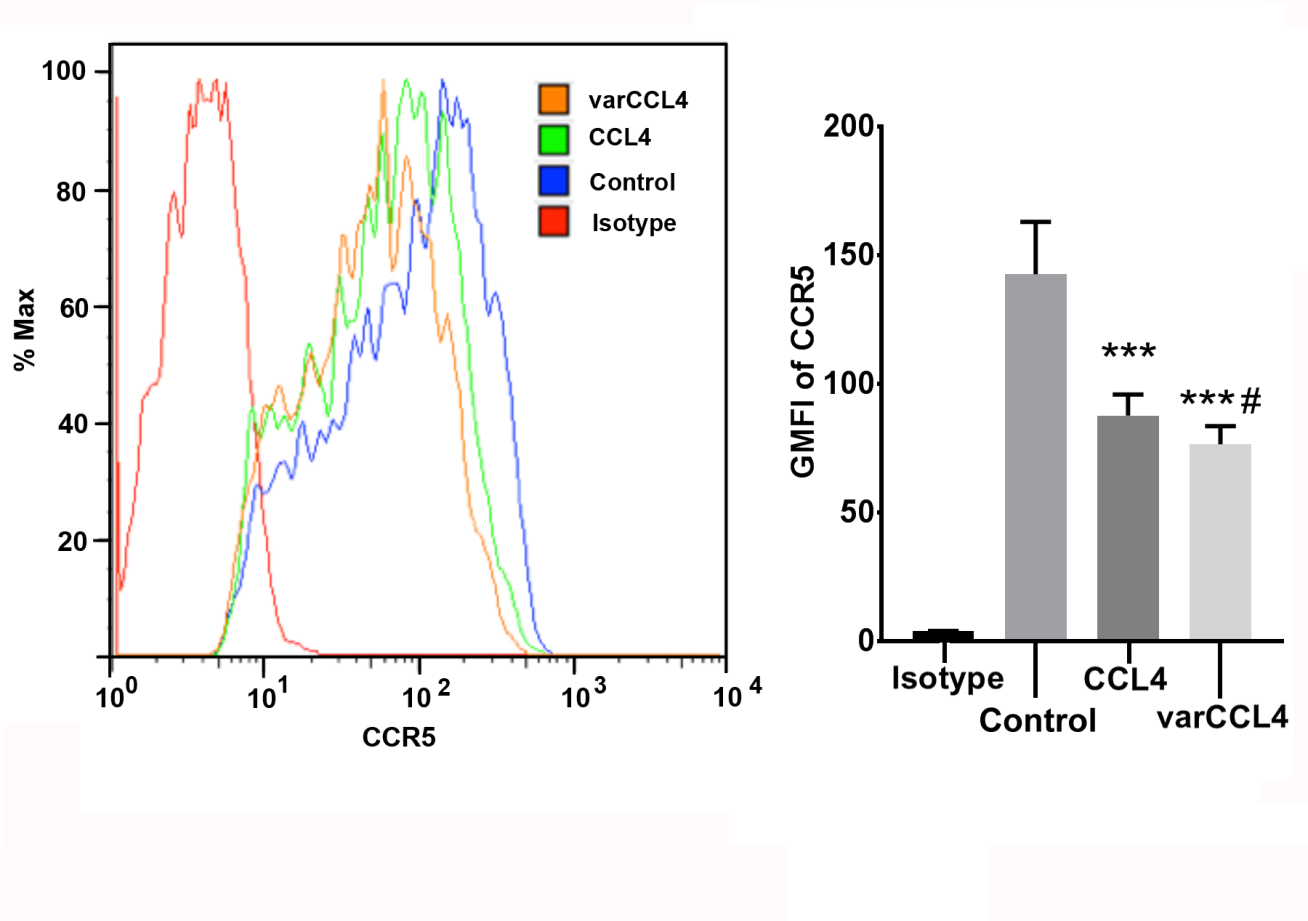
Wild type versus varCCL4 in the down-regulation of CCR5 in a 9 day mixed lymphocyte culture. Purified CD4 T-cells from mismatched donors were co-incubated for nine days and treated with WT or varCCL4 (50ng/mL) for 3 hours, surface stained with anti-CCR5 antibody and analyzed by flow cytometry (** P<0.01, *** P<0.001 in comparison with untreated control; # P<0.05 in comparison with WT CCL4).

Since the preferential target of CCL4 is CCR5-positive cells, including immature dendritic cells and activated T-cells [32], we tested the functional activity of the wild type vs varCCL4 proteins in a variety of assays. Both were able to induce a Ca2+ signal, a characteristic of CCR5 binding, and were able to block each other on sequential administration on immature dendritic cells (**Fig 3A**) and on a CCR5 positive T-cell line (**Fig 3B**). The slight difference of amplitude in Ca^2+^ signals between CCL4 and varCCL4 may suggest the variant is less potent that the WT allele. However, given the high sensitivity of this assay, both were tested in the ng/mL range where they exist mainly as dimers, making it difficult to judge the effect of aggregated varCCL4. Since a hallmark of chemokine binding to their receptors is down-regulation of the cognate receptor, we performed surface staining of CCR5^+^ cells before and after treatment with wild type and varCCL4. An equivalent amount of varCCL4 induced higher down-regulation of CCR5 in immature dendritic cells (**Fig 3C)** or in a CCR5 positive T-cell line **(Fig 3D)** than wild type CCL4. VarCCL4 also was more potent in the down-regulation of CCR5 in a mixed lymphocyte reaction (MLR), a case in which CCR5 is naturally expressed on activated T-cells (**Fig 4**). Although varCCL4 has a more potent effect on CCR5 down regulation than wild type CCL4, this effect may not be strictly related to aggregation, since the aggregation occurs at higher than physiological concentrations. We speculate that varCCL4 may cause an effect similar to the hypotension side effect caused by Malaviroc.

**Fig 5:**
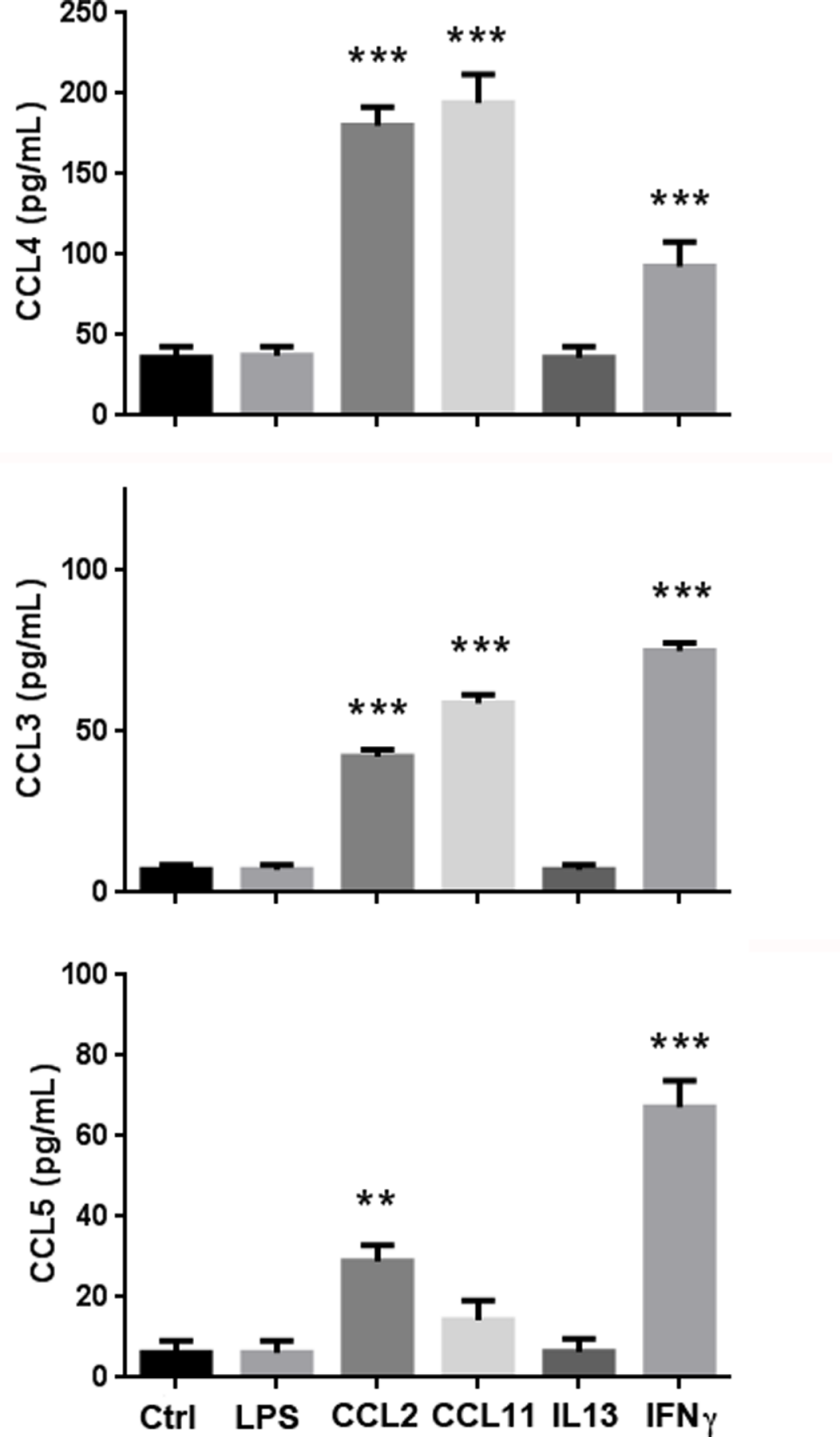
Human skeletal muscles cells secrete CCL4, CCL3 and CCL5 after treatment with cytokines and chemokines. Confluent skeletal muscle myoblasts were treated with LPS (10 ng/mL), CCL2 (20 nM), CCL11 (20 nM), IL-13 (20 nM) or IFN**γ** (100 ng/mL) for 48hrs and cytokines levels were measured using cytokine multiplex analysis. Ctrl: untreated control, n = 4 (** P<0.01, *** P<0.001 in comparison with controls).

Although many types of cells secrete CCL4 when stimulated by other cytokines or chemokines, of primary interest is the production of CCL4 by skeletal muscle cells, the main target of inflammation and pain in FM. When skeletal muscle cells were treated with a variety of inflammatory agents (including LPS and CCL11), the highest levels of CCL4 were induced by CCL11 (**Fig 5**). For comparative purposes, we also measured secretion of CCL3 and CCL5, chemokines that also bind to CCR5, from stimulated muscle cells. The results demonstrate that CCL4 levels were at least two-fold higher than either CCL3 or CCL5, emphasizing a predominant role for CCL4 in the skeletal muscle test. As previously reported by us [12], skeletal muscle cells respond to cytokine and/or chemokine stimulation by secreting their own effectors. It is possible that the production of CCL11 caused by inflammation in various sites throughout the body, may in turn, induce secretion of CCL4 in skeletal muscle, that amplify and/or prolong an inflammatory response, leading to chronic production.

### Confirmation of *MEFV* SNPs associated with FM

As mentioned in the Introduction, multiple SNPs in *MEFV* are associated with activation of the inflammasome and associated with recurrent fevers [33] via pyrin, the gene product of *MEFV*. *MEFV* variants were previously analyzed by us in 100 FM trios [13]. Although *MEFV* is located on chromosome 16, its association with the genetics of FM with the immune system supports our immune role hypothesis. We found multiple variants that collectively had significant transmission bias (p  =  0.0085), indicating a positive association between FM and variants in *MEFV*. Since variants of *MEFV* may directly affect the inflammasome [17], this analysis provides evidence for a role of the immune system in the etiology of FM. With the addition of 120 new trios, we were able to validate the association of *MEFV* variants with FM in this second cohort. We found a total of nine rare missense mutations, in combinations forming 10 distinct haplotypes, in 13 families in which we could follow 17 independent transmission events where a heterozygous parent could transmit any of the rare haplotypes to an affected child. In 13 of these 17 events (independent under the null hypothesis), the rare allele was transmitted to the affected offspring (p  =  0.029) (**Table 2**). A list of the *MEFV* variants identified are shown in **S3 Table**. When the two cohorts were combined, a total of 12 rare mutations were identified (**Table 2**). In 30 of these 39 events, the rare allele was transmitted to the affected offspring (p  =  0.008). Furthermore, among the variants analyzed in *CCL11*, *CCL4*, and *MEFV*, the overlap is no more than the product of the individual frequencies (data not shown), suggesting each are independently associated with FM.

**Table 2:** Transmission analysis of *MEFV* in cohort of 120 fibromyalgia trios^1^.

**variant**	**proband**	**mother**	**father**	**transmitted**	**untransmitted**
E148Q	FM647 het	wt	het	1	0
	FM624 het	het	wt	1	0
L110P/E148Q	FM826 het	het	wt	1	0
	FM835 wt	wt	het	0	1
P369S/R408Q	FM627 wt	het	wt	0	1
	FM635 het	wt	het	1	0
	FM688 het	wt	het	1	0
L110P/E148Q/P369S	FM887 het	wt	het	1	0
E148Q/P369S/R408Q	FM662 het	wt	het	1	0
E526G	FM944 het	het	wt	1	0
I591T	FM699 wt	het	wt	0	1
	FM956 het	wt	het	1	0
	FM739 het	wt	het	1	0
K695R	FM699 wt	het	wt	0	1
	FM956 het	wt	het	1	0
V726A	FM841 het	het	wt	1	0
A744S	FM736 het	het	wt	1	0
Total				13	4
					
** **	**Cohort 1**	**Cohort 2**		**Total**	
**Number of trios**	100	120		220	
**Probands with SNP**	15	13		28	
**Transmitted**	17	13		30	
**Not transmitted**	5	4		9	
**P value**	0.0085	0.029		0.008	

1 Upper: The size of the cohort was 120 trios (wt = wild type, het = heterozygous). Lower: Combined cohorts of 100 trios previously reported together with new cohort of 120 (var = variant SNP).

## Discussion

Although the cause of FM is unknown and there are no definitive laboratory tests, many patients report a triggering event such as trauma or a chronic inflammatory condition. Trauma that causes sterile inflammation and chronic inflammation have in common the triggering of the immune response [34]. While high levels of chemokines have been reported in the blood [12] and CNS [35] of FM patients, the prevailing hypothesis centers on disturbances in pain pathways [36]. Given the high incidence within families, clues to its origin may lie in genetic analyses. Using a FM family GWAS that identified a region of chromosome 17 which included a cluster of 18 chemokine genes as a starting point, we tested the hypothesis that the pathogenesis of FM is immune-related. Out of four candidate SNPs in four chemokine genes, a variant allele in *CCL11* found in 36.8% of 220 FM cases had a statistically significant TDT (p = 0.0074). Functional studies revealed that the resulting protein from the variant affected expression due to its location at the precise point of cleavage of the signal peptide from pre-pro-CCL11. Since CCL11 levels are statistically elevated in most FM patients, this result suggests that while the elevated expression of CCL11 is a common event, the inability to generate a robust CCL11 response predisposes up to 36% of patients with a higher likelihood of FM. While we have no obvious explanation of why this is the case, it may suggest that high expression of CCL11 in response to an inflammatory trigger is a compensatory feature of FM since the CCL11 SNP prevents a robust response. With further validation studies, it is possible that SNP analysis of rs1129844 in *CCL11* can be used as a risk factor for FM. This study provides evidence that rs1129844 in *CCL11* may be a useful marker for FM and that the high frequency of this SNP in FM patients (36.8%) argues for an underlying immune connection.

While SNP rs1719152 in *CCL4* failed to give a positive TDT and may not be causal, additional functional studies demonstrated the induction of CCL4 from skeletal muscle cells treated with CCL11, perhaps explaining a cause and effect relationship between the two chemokines at the level of skeletal muscle. CCL4 has long been associated with neuropathic pain [37], while muscle pain in fibromyalgia is associated with high levels of the related chemokine CCL2 [12, 38, 39]. The further observation that down-regulation of CCR5, the cognate receptor for CCL4, causes hypotension is an interesting correlation, since many FM patients report episodes of orthostatic hypotension [28, 40]. We speculate that there is a relationship between CCL11 and CCL4 in FM that may account for symptomology.

If a chronic inflammatory condition, whether sterile or due to a chronic condition such as IBS, is a trigger for FM, then chronic activation of the immune system may account for many of the symptoms. In earlier work, we speculated that the inflammasome, known to be triggered by both PAMPs and DAMPs [33], may be chronically activated in FM patients with causative SNPs in the *MEFV* gene. While periodic fevers require causative SNPs on both alleles of *MEFV*, we speculated that a SNP on one allele may lead to activation of the inflammasome in the presence of other environmental factors. Since our current study involved a second cohort of 100 FM families, there was an opportunity to reexamine the contribution of *MEFV* SNPs to FM. Not unexpectedly, we found a significant TDT in the second cohort (p = 0.012), and when combined an overall TDT with p = 0.008. Since only 12% of the combined cohort had *MEFV* SNPs, we conclude that the different aspects of the immune system may contribute to common symptoms in FM, suggesting that different genetic tests with appropriate approaches to treatment may be warranted.

In summary, we present evidence at both the genetic and functional level that the immune system may be involved in FM in roughly half of a cohort of 220 FM patients for which SNPs in *CCL11* and *MEFV* gave significant TDTs. Considering that activation of the immune system is often associated with neurological systems such as pain, the involvement of the immune system in FM does not rule out the prevailing hypothesis that FM is predominantly a pain syndrome. With this in mind, further studies on larger number of patients may help to validate the link between pain and the immune system in FM.

## Supporting information

S1 Table SNPs in chromosome 17 (17p13.3 to 17q25.3) found in 10% or more of a fibromyalgia cohort of 100. (DOCX)

S2 Table Transmission analysis of CCL11 variant -146 C>T in 120 FM trios. (DOCX)

S3 Table *MEFV* SNPs identified in 220 fibromyalgia probands. (DOCX)

S1 Fig Example of sequence analysis of a FM patient heterozygous for rs1129844 on *CCL11*. (DOCX)

S2 Fig Protein size profiles of wild type CCL4 and varCCL4. (DOCX)
