# Supplementary material for: SNPs in inflammatory genes CCL11, CCL4 and MEFV in a fibromyalgia family study
Source: PLoS One. 2018 Jun 21;13(6):e0198625. doi: 10.1371/journal.pone.0198625 (PMC6013222; doi:10.1371/journal.pone.0198625)
Supplement: S1 Table — (DOCX) [file pone.0198625.s001.docx]

**S1 Table. SNPs in chromosome 17 (17p13.3 to 17q25.3) found in 10% or more of a fibromyalgia cohort of 100^1^.**

|  |  |  |  |  |  |  | **FM patients** | | | | |  |
| --- | --- | --- | --- | --- | --- | --- | --- | --- | --- | --- | --- | --- |
|  | **Gene** | **Position** | **Ref > Var** | **RSID** | **AA Change** | **AA Pos** | **Ref** | **Hetero** | **Homo** | **Var** | **Allele Freq** | **ExAc Freq** |
| 1 | DNAH9 | 11523082 | A>G | 9892256 | GLN_ARG | 445 | 0 | 0 | 100 | 200 | 100.00% | 99.99% |
| 2 | DNAH9 | 11650958 | G>A | 61743635 | ARG_LYS | 2162 | 82 | 18 | 0 | 18 | 9.00% | 10.07% |
| 3 | DNAH9 | 11651057 | A>G | 3744581 | ASN_SER | 2195 | 62 | 29 | 9 | 47 | 23.50% | 23.19% |
| 4 | DNAH9 | 11672607 | G>T | 61744697 | VAL_LEU | 2505 | 79 | 19 | 2 | 23 | 11.50% | 15.05% |
| 5 | DNAH9 | 11835331 | G>A | 17612861 | ASP_ASN | 4036 | 55 | 34 | 11 | 56 | 28.00% | 33.68% |
| 6 | DNAH9 | 11865462 | G>A | 1990236 | MET_ILE | 4374 | 74 | 22 | 4 | 30 | 15.00% | 18.25% |
| 7 | ARHGAP44 | 12862029 | C>T | 3213689 | none |  | 80 | 19 | 1 | 21 | 10.50% | 9.79% |
| 8 | ELAC2 | 12899902 | C>T | 5030739 | ALA_THR | 501 | 88 | 12 | 0 | 12 | 6.00% | 3.98% |
| 9 | ELAC2 | 12915009 | G>A | 4792311 | SER_LEU | 217 | 53 | 36 | 11 | 58 | 29.00% | 30.58% |
| 10 | COX10 | 13980058 | A>T | 2230351 | THR_SER | 62 | 89 | 11 | 0 | 11 | 5.50% | 7.59% |
| 11 | COX10 | 13980350 | G>A | 2072279 | ARG_GLN | 159 | 31 | 43 | 26 | 95 | 47.50% | 50.54% |
| 12 | CDRT15 | 14139891 | A>C | 62052131 | LEU_ARG | 87 | 0 | 0 | 87 | 174 | 100.00% | 99.89% |
| 13 | CDRT15 | 14140081 | G>A | 11651890 | ARG_stop | 24 | 37 | 13 | 0 | 13 | 13.00% | 10.60% |
| 14 | TEKT3 | 15217437 | C>G | 230898 | GLY_ALA | 282 | 29 | 46 | 25 | 96 | 48.00% | 47.23% |
| 15 | TEKT3 | 15234895 | C>T | 7226363 | ARG_HIS | 3 | 60 | 34 | 6 | 46 | 23.00% | 24.03% |
| 16 | CDRT4_TVP23C-CDRT4 | 15341183 | A>C | 2954759 | HIS_GLN | 122 | 75 | 23 | 2 | 27 | 13.50% | 10.26% |
| 17 | TVP23C_TVP23C-CDRT4 | 15406197 | A>T | 12150518 | LEU_GLN | 271 | 86 | 13 | 1 | 15 | 7.50% | 6.61% |
| 18 | CDRT1 | 15492410 | G>A | 76213106 | ALA_VAL | 713 | 51 | 33 | 6 | 45 | 25.00% | 9.89% |
| 19 | CDRT1 | 15492426 | C>T | 28445278 | VAL_MET | 708 | 5 | 20 | 60 | 140 | 82.35% | 54.19% |
| 20 | CDRT1 | 15496727 | T>G | 79385100 | ASN_HIS | 644 | 81 | 14 | 5 | 24 | 12.00% | 13.38% |
| 21 | CDRT1 | 15496730 | A>G | 8078150 | PHE_LEU | 643 | 57 | 34 | 9 | 52 | 26.00% | 25.46% |
| 22 | CDRT1 | 15510888 | G>A | 62070401 | THR_MET | 411 | 33 | 15 | 2 | 19 | 19.00% | 17.81% |
| 23 | CDRT1 | 15510988 | T>C | 62070402 | ASN_ASP | 378 | 33 | 15 | 2 | 19 | 19.00% | 17.73% |
| 24 | CDRT1 | 15517237 | G>C | 62070406 | LEU_VAL | 261 | 64 | 27 | 9 | 45 | 22.50% | 19.09% |
| 25 | CDRT1 | 15517284 | G>C | 200934978 | ALA_GLY | 245 | 72 | 17 | 10 | 37 | 18.69% | 3.04% |
| 26 | TRIM16 | 15531942 | C>A | 1060903 | GLY_VAL | 561 | 62 | 31 | 6 | 43 | 21.72% | 26.00% |
| 27 | TRIM16 | 15532147 | G>A | 3174720 | ARG_TRP | 493 | 88 | 12 | 0 | 12 | 6.00% | 5.25% |
| 28 | TRIM16 | 15554561 | C>A | 2074890 | GLU_ASP | 121 | 86 | 14 | 0 | 14 | 7.00% | 4.54% |
| 29 | ZNF286A | 15611495 | T>C | 3760299 | TYR_HIS | 90 | 40 | 48 | 12 | 72 | 36.00% | 36.75% |
| 30 | TBC1D26 | 15640815 | A>G | 201215351 | HIS_ARG | 59 | 36 | 8 | 5 | 18 | 18.37% | 4.22% |
| 31 | TBC1D26 | 15644506 | C>T | 11650318 | ALA_VAL | 206 | 48 | 37 | 15 | 67 | 33.50% | 39.90% |
| 32 | TBC1D26 | 15645289 | G>A | 17855672 | GLY_SER | 234 | 48 | 37 | 15 | 67 | 33.50% | 39.90% |
| 33 | NCOR1 | 15973844 | A>T | 12942295 | none |  | 26 | 47 | 27 | 101 | 50.50% | 53.64% |
| 34 | NCOR1 | 16068340 | C>T | 76780359 | GLU_LYS | 82 | 0 | 53 | 0 | 53 | 50.00% | 41.61% |
| 35 | NCOR1 | 16068343 | G>A | 78230791 | ARG_stop | 81 | 0 | 52 | 0 | 52 | 50.00% | 41.73% |
| 36 | NCOR1 | 16068377 | C>G | 200020868 | LYS_ASN | 69 | 0 | 51 | 0 | 51 | 50.00% | 41.83% |
| 37 | NCOR1 | 16068396 | G>A | 150910818 | SER_LEU | 63 | 0 | 52 | 0 | 52 | 50.00% | 47.04% |
| 38 | NCOR1 | 16068463 | C>T | 74453660 | GLY_ARG | 41 | 20 | 32 | 0 | 32 | 30.77% | 35.09% |
| 39 | NCOR1 | 16097870 | C>A | 76145228 | GLY_VAL | 5 | 56 | 44 | 0 | 44 | 22.00% | 37.61% |
| 40 | C17orf76-AS1_FAM211A | 16347325 | T>C | 61745139 | ARG_GLY | 166 | 85 | 14 | 1 | 16 | 8.00% | 13.49% |
| 41 | ZNF624 | 16527795 | C>A | 8065506 | LYS_ASN | 135 | 45 | 45 | 10 | 65 | 32.50% | 27.83% |
| 42 | TNFRSF13B | 16842991 | G>A | 34562254 | PRO_LEU | 251 | 82 | 17 | 1 | 19 | 9.50% | 11.39% |
| 43 | MPRIP | 17046024 | C>A | 3744137 | PRO_GLN | 327 | 21 | 51 | 28 | 107 | 53.50% | 52.84% |
| 44 | FLCN | 17122327 | G>A | 8065832 | none |  | 29 | 42 | 29 | 100 | 50.00% | 49.37% |
| 45 | COPS3 | 17179481 | G>A | 4985761 | none |  | 27 | 45 | 28 | 101 | 50.50% | 49.75% |
| 46 | SMCR9 | 17326363 | G>A | 35590625 | VAL_ILE | 60 | 53 | 38 | 9 | 56 | 28.00% | 37.40% |
| 47 | SMCR9 | 17326604 | C>T | 7225976 | ALA_VAL | 140 | 44 | 42 | 14 | 70 | 35.00% | 46.07% |
| 48 | PEMT | 17409560 | C>T | 7946 | VAL_MET | 190 | 4 | 33 | 40 | 113 | 73.38% | 73.35% |
| 49 | PEMT | 17425631 | C>T | 897453 | VAL_ILE | 73 | 35 | 48 | 17 | 82 | 41.00% | 45.75% |
| 50 | RAI1 | 17696531 | G>C | 3803763 | GLY_ALA | 90 | 45 | 46 | 9 | 64 | 32.00% | 32.12% |
| 51 | RAI1 | 17696755 | C>A | 11649804 | PRO_THR | 165 | 45 | 46 | 9 | 64 | 32.00% | 30.47% |
| 52 | LRRC48 | 17896205 | C>T | 4584886 | ARG_TRP | 191 | 47 | 41 | 12 | 65 | 32.50% | 32.87% |
| 53 | DRG2 | 18003671 | T>G | 2272570 | none |  | 1 | 15 | 84 | 183 | 91.50% | 91.56% |
| 54 | MYO15A | 18023897 | G>A | 2955365 | ALA_THR | 595 | 42 | 35 | 6 | 47 | 28.31% | 41.84% |
| 55 | MYO15A | 18024266 | T>G | 2955367 | TRP_GLY | 718 | 22 | 20 | 6 | 32 | 33.33% | 37.78% |
| 56 | MYO15A | 18046898 | T>C | 854777 | CYS_ARG | 1977 | 10 | 27 | 47 | 121 | 72.02% | 78.53% |
| 57 | MYO15A | 18047189 | G>A | 2272571 | GLY_ARG | 2018 | 77 | 20 | 3 | 26 | 13.00% | 18.77% |
| 58 | MYO15A | 18057167 | A>T | 712270 | TYR_PHE | 2682 | 38 | 39 | 17 | 73 | 38.83% | 41.27% |
| 59 | MYO15A | 18057215 | C>G | 9916193 | none |  | 59 | 30 | 4 | 38 | 20.43% | 22.02% |
| 60 | LLGL1 | 18137141 | A>G | 2290505 | SER_GLY | 148 | 8 | 37 | 55 | 147 | 73.50% | 74.63% |
| 61 | MIEF2 | 18167397 | G>T | 3889402 | ALA_SER | 204 | 77 | 20 | 2 | 24 | 12.12% | 14.14% |
| 62 | MIEF2 | 18167684 | G>A | 12603700 | GLY_GLU | 324 | 78 | 21 | 1 | 23 | 11.50% | 9.68% |
| 63 | SMCR8 | 18220674 | C>T | 8080966 | PRO_LEU | 524 | 50 | 43 | 7 | 57 | 28.50% | 30.61% |
| 64 | SMCR8 | 18220770 | G>A | 1563632 | ARG_HIS | 556 | 11 | 43 | 46 | 135 | 67.50% | 67.40% |
| 65 | SMCR8 | 18221010 | A>G | 12449313 | ASN_SER | 636 | 54 | 37 | 9 | 55 | 27.50% | 25.91% |
| 66 | SHMT1 | 18232096 | G>A | 1979277 | LEU_PHE | 474 | 52 | 41 | 7 | 55 | 27.50% | 42.26% |
| 67 | EVPLL | 18284262 | G>A | 570145 | SER_ASN | 4 | 17 | 45 | 38 | 121 | 60.50% | 69.16% |
| 68 | LGALS9C | 18380185 | G>C | 79350397 | CYS_SER | 6 | 80 | 11 | 1 | 13 | 7.07% | 8.59% |
| 69 | LGALS9C | 18396007 | C>G | 1725650 | none |  | 61 | 20 | 2 | 24 | 14.46% | 3.25% |
| 70 | TBC1D28 | 18542519 | T>G | 74452761 | GLU_ALA | 56 | 10 | 40 | 4 | 48 | 44.44% | 40.31% |
| 71 | ZNF286B | 18565350 | G>C | 9912644 | THR_SER | 490 | 38 | 55 | 7 | 69 | 34.50% | 35.19% |
| 72 | ZNF286B | 18565423 | G>A | 9912852 | PRO_SER | 466 | 37 | 51 | 12 | 75 | 37.50% | 38.72% |
| 73 | TRIM16L | 18630995 | G>A | 8075739 | ARG_LYS | 42 | 36 | 50 | 14 | 78 | 39.00% | 39.77% |
| 74 | FBXW10 | 18647625 | T>A | 74502778 | ILE_ASN | 23 | 7 | 24 | 19 | 62 | 62.00% | 67.19% |
| 75 | FBXW10 | 18653070 | G>A | 9895749 | GLU_LYS | 236 | 35 | 53 | 11 | 75 | 37.88% | 41.22% |
| 76 | FBXW10 | 18653145 | C>G | 200535885 | LEU_VAL | 261 | 67 | 33 | 0 | 33 | 16.50% | 21.12% |
| 77 | FBXW10 | 18653188 | G>T | 139431164 | ARG_LEU | 275 | 84 | 14 | 2 | 18 | 9.00% | 7.80% |
| 78 | FBXW10 | 18671961 | C>T | 144931466 | ARG_CYS | 607 | 77 | 15 | 1 | 17 | 9.14% | 7.55% |
| 79 | TVP23B | 18694277 | G>A | 61075345 | GLY_GLU | 55 | 2 | 25 | 68 | 161 | 84.74% | 84.29% |
| 80 | SLC5A10_FAM83G | 18882991 | T>A | 2472714 | none |  | 0 | 1 | 99 | 199 | 99.50% | 99.99% |
| 81 | EPN2 | 19232078 | T>C | 6587220 | VAL_ALA | 116 | 0 | 3 | 97 | 197 | 98.50% | 98.78% |
| 82 | B9D1 | 19247075 | G>A | 4924987 | HIS_TYR | 187 | 8 | 19 | 73 | 165 | 82.50% | 81.07% |
| 83 | RNF112 | 19319015 | C>T | 1295329 | none |  | 5 | 44 | 51 | 146 | 73.00% | 69.70% |
| 84 | SLC47A1 | 19454733 | G>A | 2247436 | none |  | 59 | 35 | 6 | 47 | 23.50% | 18.06% |
| 85 | ALDH3A1 | 19642952 | G>C | 2228100 | PRO_ALA | 329 | 58 | 35 | 7 | 49 | 24.50% | 26.07% |
| 86 | ALDH3A1 | 19645938 | A>C | 887241 | SER_ALA | 134 | 10 | 48 | 42 | 132 | 66.00% | 66.35% |
| 87 | ULK2 | 19713740 | C>T | 150122 | VAL_MET | 370 | 1 | 9 | 90 | 189 | 94.50% | 96.68% |
| 88 | AKAP10 | 19812541 | T>C | 203462 | ILE_VAL | 646 | 30 | 45 | 25 | 95 | 47.50% | 37.90% |
| 89 | AKAP10 | 19835112 | C>T | 61749865 | none |  | 68 | 30 | 1 | 32 | 16.16% | 11.89% |
| 90 | AKAP10 | 19861458 | C>T | 2108978 | ARG_HIS | 249 | 30 | 45 | 25 | 95 | 47.50% | 37.82% |
| 91 | SPECC1 | 20108184 | C>G | 9908032 | SER_ARG | 274 | 70 | 29 | 1 | 31 | 15.50% | 12.52% |
| 92 | SPECC1 | 20108239 | A>T | 2703806 | MET_LEU | 293 | 15 | 51 | 34 | 119 | 59.50% | 57.68% |
| 93 | SPECC1 | 20160925 | C>T | 3751979 | none |  | 57 | 40 | 3 | 46 | 23.00% | 17.75% |
| 94 | LGALS9B | 20370767 | G>C | 4985834 | SER_CYS | 6 | 7 | 9 | 37 | 83 | 78.30% | 90.69% |
| 95 | CCDC144NL | 20768730 | A>G | 4605228 | stop_GLN | 222 | 21 | 29 | 0 | 29 | 29.00% | 4.05% |
| 96 | CCDC144NL | 20768744 | G>T | 62066974 | SER_TYR | 217 | 29 | 22 | 0 | 22 | 21.57% | 1.01% |
| 97 | CCDC144NL | 20768788 | G>T | 78365129 | HIS_GLN | 202 | 14 | 38 | 0 | 38 | 36.54% | 2.22% |
| 98 | CCDC144NL | 20768816 | C>T | 73298040 | CYS_TYR | 193 | 14 | 38 | 0 | 38 | 36.54% | 7.54% |
| 99 | CCDC144NL | 20769862 | T>A | 79433424 | GLY | 190 | 0 | 60 | 2 | 64 | 51.61% | 25.96% |
| 100 | CCDC144NL | 20769866 | G>A | 79678106 | PRO_LEU | 189 | 0 | 60 | 3 | 66 | 52.38% | 29.63% |
| 101 | CCDC144NL | 20769896 | G>T | 79843086 | THR_ASN | 179 | 0 | 59 | 6 | 71 | 54.62% | 45.97% |
| 102 | CCDC144NL | 20769899 | G>T | 79930314 | SER_stop | 178 | 0 | 58 | 6 | 70 | 54.69% | 45.88% |
| 103 | CCDC144NL | 20769954 | T>G | 77065992 | THR_PRO | 160 | 1 | 59 | 2 | 63 | 50.81% | 26.32% |
| 104 | CCDC144NL | 20769968 | G>A | 78792130 | THR_MET | 155 | 1 | 62 | 4 | 70 | 52.24% | 24.09% |
| 105 | CCDC144NL | 20769995 | C>T | 78132860 | GLY_GLU | 146 | 0 | 61 | 3 | 67 | 52.34% | 26.99% |
| 106 | CCDC144NL | 20770002 | C>A | 80298820 | ALA_SER | 144 | 0 | 61 | 2 | 65 | 51.59% | 28.21% |
| 107 | CCDC144NL | 20770003 | C>G | 76947654 | GLN_HIS | 143 | 0 | 61 | 2 | 65 | 51.59% | 29.39% |
| 108 | CCDC144NL | 20799119 | A>G | 2279263 | LEU_PRO | 72 | 11 | 27 | 12 | 51 | 51.00% | 48.08% |
| 109 | MAP2K3 | 21201719 | T>C | 8076154 | none |  | 0 | 100 | 0 | 100 | 50.00% | 50.00% |
| 110 | MAP2K3 | 21202191 | C>A | 33911218 | PRO_THR | 11 | 0 | 100 | 0 | 100 | 50.00% | 50.00% |
| 111 | MAP2K3 | 21202237 | G>C | 36047035 | ARG_THR | 26 | 0 | 100 | 0 | 100 | 50.00% | 50.00% |
| 112 | MAP2K3 | 21203893 | T>C | 34105301 | SER_PRO | 39 | 3 | 97 | 0 | 97 | 48.50% | 49.96% |
| 113 | MAP2K3 | 21203941 | G>A | 2305873 | ALA_THR | 55 | 55 | 44 | 0 | 44 | 22.22% | 20.61% |
| 114 | MAP2K3 | 21204187 | G>T | 56067280 | ARG_LEU | 65 | 0 | 100 | 0 | 100 | 50.00% | 49.99% |
| 115 | MAP2K3 | 21204192 | C>T | 56216806 | ARG_TRP | 67 | 0 | 100 | 0 | 100 | 50.00% | 49.99% |
| 116 | MAP2K3 | 21204210 | C>T | 55796947 | GLN_stop | 73 | 0 | 100 | 0 | 100 | 50.00% | 49.99% |
| 117 | MAP2K3 | 21204308 | G>T | 55777930 | none |  | 0 | 100 | 0 | 100 | 50.00% | 49.98% |
| 118 | MAP2K3 | 21207834 | C>T | 58609466 | THR_MET | 193 | 51 | 49 | 0 | 49 | 24.50% | 29.43% |
| 119 | MAP2K3 | 21215557 | G>A | 35206134 | ARG_HIS | 264 | 0 | 100 | 0 | 100 | 50.00% | 49.99% |
| 120 | MAP2K3 | 21217513 | G>A | 2363198 | VAL_MET | 310 | 0 | 100 | 0 | 100 | 50.00% | 50.00% |
| 121 | KCNJ12 | 21318698 | C>T | 1657738 | SER_LEU | 15 | 1 | 99 | 0 | 99 | 49.50% | 49.91% |
| 122 | KCNJ12 | 21318760 | G>T | 74880280 | VAL_LEU | 36 | 78 | 22 | 0 | 22 | 11.00% | 9.93% |
| 123 | KCNJ12 | 21318770 | G>A | 3752033 | ARG_GLN | 39 | 47 | 53 | 0 | 53 | 26.50% | 34.43% |
| 124 | KCNJ12 | 21318773 | G>A | 3752034 | ARG_HIS | 40 | 45 | 55 | 0 | 55 | 27.50% | 34.30% |
| 125 | KCNJ12 | 21318782 | G>A | 78117732 | ARG_HIS | 43 | 3 | 97 | 0 | 97 | 48.50% | 48.52% |
| 126 | KCNJ12 | 21318821 | A>C | 1714865 | GLU_ALA | 56 | 41 | 59 | 0 | 59 | 29.50% | 36.93% |
| 127 | KCNJ12 | 21318867 | G>A | 73979893 | MET_ILE | 71 | 2 | 98 | 0 | 98 | 49.00% | 49.24% |
| 128 | KCNJ12 | 21318952 | A>G | 8076599 | ILE_VAL | 100 | 0 | 100 | 0 | 100 | 50.00% | 50.00% |
| 129 | KCNJ12 | 21319007 | G>A | 1657740 | ARG_GLN | 118 | 0 | 100 | 0 | 100 | 50.00% | 49.94% |
| 130 | KCNJ12 | 21319069 | G>A | 76265595 | GLU_LYS | 139 | 3 | 97 | 0 | 97 | 48.50% | 48.97% |
| 131 | KCNJ12 | 21319079 | C>A | 76518282 | THR_ASN | 142 | 32 | 68 | 0 | 68 | 34.00% | 34.27% |
| 132 | KCNJ12 | 21319087 | G>A | 75029097 | GLY_SER | 145 | 2 | 98 | 0 | 98 | 49.00% | 49.02% |
| 133 | KCNJ12 | 21319121 | C>T | 1714864 | PRO_LEU | 156 | 0 | 100 | 0 | 100 | 50.00% | 49.99% |
| 134 | KCNJ12 | 21319171 | G>A | 73313922 | ASP_ASN | 173 | 0 | 100 | 0 | 100 | 50.00% | 47.31% |
| 135 | KCNJ12 | 21319208 | C>T | 73979896 | ALA_VAL | 185 | 1 | 99 | 0 | 99 | 49.50% | 46.34% |
| 136 | KCNJ12 | 21319230 | G>C | 1657742 | GLN_HIS | 192 | 1 | 99 | 0 | 99 | 49.50% | 49.74% |
| 137 | KCNJ12 | 21319285 | C>T | 72846667 | LEU_PHE | 211 | 2 | 98 | 0 | 98 | 49.00% | 49.36% |
| 138 | KCNJ12 | 21319369 | G>A | 77048459 | GLU_LYS | 239 | 39 | 61 | 0 | 61 | 30.50% | 25.49% |
| 139 | KCNJ12 | 21319399 | A>G | 4985866 | ILE_VAL | 249 | 48 | 52 | 0 | 52 | 26.00% | 33.89% |
| 140 | KCNJ12 | 21319436 | G>A | 77270326 | ARG_HIS | 261 | 7 | 93 | 0 | 93 | 46.50% | 17.32% |
| 141 | KCNJ12 | 21319439 | T>G | 76684759 | ILE_SER | 262 | 12 | 88 | 0 | 88 | 44.00% | 18.74% |
| 142 | KCNJ12 | 21319519 | G>C | 78113532 | GLU_GLN | 289 | 11 | 89 | 0 | 89 | 44.50% | 27.63% |
| 143 | KCNJ12 | 21319523 | C>T | 77987694 | THR_MET | 290 | 12 | 88 | 0 | 88 | 44.00% | 28.43% |
| 144 | KCNJ12 | 21319543 | G>A | 80335301 | VAL_ILE | 297 | 8 | 92 | 0 | 92 | 46.00% | 32.31% |
| 145 | KCNJ12 | 21319560 | G>T | 74801394 | MET_ILE | 302 | 4 | 96 | 0 | 96 | 48.00% | 39.76% |
| 146 | KCNJ12 | 21319682 | C>T | 80203231 | SER_LEU | 343 | 4 | 96 | 0 | 96 | 48.00% | 48.46% |
| 147 | KCNJ12 | 21319786 | G>A | 78547883 | GLU_LYS | 378 | 0 | 100 | 0 | 100 | 50.00% | 49.14% |
| 148 | KCNJ12 | 21319868 | G>T | 73979902 | SER_ILE | 405 | 2 | 98 | 0 | 98 | 49.00% | 48.33% |
| 149 | KCNJ12 | 21319943 | A>G | 5021699 | GLU_GLY | 430 | 2 | 98 | 0 | 98 | 49.00% | 49.74% |
| 150 | WSB1 | 25628820 | T>C | 6561 | LEU_SER | 16 | 35 | 46 | 19 | 84 | 42.00% | 42.25% |
| 151 | WSB1 | 25630388 | T>C | 2270761 | none |  | 35 | 46 | 19 | 84 | 42.00% | 42.34% |
| 152 | KSR1 | 25909816 | C>T | 2293180 | ALA_VAL | 85 | 79 | 20 | 1 | 22 | 11.00% | 12.22% |
| 153 | LGALS9 | 25958304 | G>A | 3751093 | GLY_SER | 5 | 67 | 30 | 3 | 36 | 18.00% | 21.14% |
| 154 | LGALS9 | 25970633 | G>A | 361497 | GLY_GLU | 176 | 31 | 19 | 2 | 23 | 22.12% | 24.88% |
| 155 | LGALS9 | 25970642 | A>T | 361498 | GLN_LEU | 179 | 33 | 16 | 2 | 20 | 19.61% | 23.48% |
| 156 | NOS2 | 26092555 | G>A | 2297512 | none |  | 17 | 37 | 33 | 103 | 59.20% | 60.85% |
| 157 | NOS2 | 26096597 | G>A | 2297518 | SER_LEU | 608 | 65 | 33 | 2 | 37 | 18.50% | 19.89% |
| 158 | POLDIP2 | 26684449 | G>C | 4795429 | PRO_ALA | 9 | 0 | 0 | 52 | 104 | 100.00% | 99.98% |
| 159 | SEBOX | 26691321 | A>G | 9910163 | LEU_SER | 207 | 9 | 29 | 62 | 153 | 76.50% | 76.95% |
| 160 | VTN | 26694861 | G>A | 704 | THR_MET | 400 | 27 | 52 | 21 | 94 | 47.00% | 47.58% |
| 161 | SARM1 | 26699121 | G>C | 7212814 | ARG_PRO | 23 | 0 | 0 | 95 | 190 | 100.00% | 100.00% |
| 162 | SARM1 | 26708304 | T>G | 71373647 | SER_ALA | 185 | 0 | 0 | 100 | 200 | 100.00% | NA |
| 163 | SLC13A2 | 26824156 | A>G | 11567842 | ILE_VAL | 599 | 41 | 48 | 11 | 70 | 35.00% | 35.07% |
| 164 | FOXN1 | 26851602 | C>T | 2071587 | ARG_CYS | 69 | 84 | 15 | 1 | 17 | 8.50% | 8.05% |
| 165 | FOXN1 | 26861877 | C>T | 61749867 | PRO_SER | 430 | 89 | 10 | 1 | 12 | 6.00% | 4.67% |
| 166 | FOXN1 | 26864302 | G>C | 532648 | ALA_PRO | 599 | 51 | 38 | 11 | 60 | 30.00% | 29.33% |
| 167 | KIAA0100 | 26947652 | G>A | 688536 | none |  | 59 | 37 | 4 | 45 | 22.50% | 23.37% |
| 168 | KIAA0100 | 26955330 | A>C | 12602520 | VAL_GLY | 1516 | 77 | 22 | 1 | 24 | 12.00% | 8.66% |
| 169 | FLOT2 | 27210253 | T>C | 6505095 | none |  | 45 | 43 | 12 | 67 | 33.50% | 32.97% |
| 170 | DHRS13 | 27225586 | A>T | 4795472 | LEU_GLN | 336 | 82 | 17 | 1 | 19 | 9.50% | 13.66% |
| 171 | SEZ6 | 27284443 | A>G | 12941884 | MET_THR | 806 | 71 | 26 | 3 | 32 | 16.00% | 13.02% |
| 172 | SEZ6 | 27286851 | T>C | 1976165 | THR_ALA | 546 | 55 | 37 | 8 | 53 | 26.50% | 25.96% |
| 173 | MYO18A | 27438469 | G>A | 8076604 | ALA_VAL | 958 | 28 | 43 | 27 | 97 | 49.49% | 46.59% |
| 174 | ABHD15 | 27889986 | T>C | 542939 | THR_ALA | 334 | 11 | 36 | 53 | 142 | 71.00% | 65.25% |
| 175 | ABHD15 | 27893893 | C>A | 62070806 | ARG_LEU | 31 | 39 | 10 | 1 | 12 | 12.00% | 10.44% |
| 176 | SSH2 | 27959903 | G>A | 2289629 | SER_LEU | 743 | 50 | 42 | 8 | 58 | 29.00% | 33.30% |
| 177 | EFCAB5 | 28296327 | T>G | 9897794 | LEU_VAL | 181 | 24 | 45 | 31 | 107 | 53.50% | 50.77% |
| 178 | EFCAB5 | 28320248 | T>A | 4795524 | ILE_LYS | 222 | 0 | 8 | 92 | 192 | 96.00% | 97.51% |
| 179 | BLMH | 28576076 | T>C | 1050565 | ILE_VAL | 443 | 45 | 49 | 6 | 61 | 30.50% | 32.48% |
| 180 | TBC1D29 | 28887134 | G>A | 111780165 | none |  | 85 | 15 | 0 | 15 | 7.50% | 7.80% |
| 181 | CRLF3 | 29111368 | A>G | 11867457 | LEU_PRO | 389 | 70 | 26 | 4 | 34 | 17.00% | 17.44% |
| 182 | ATAD5 | 29161202 | A>T | 9910051 | THR_SER | 35 | 71 | 24 | 5 | 34 | 17.00% | 11.54% |
| 183 | ATAD5 | 29161358 | C>T | 3816780 | PRO_SER | 87 | 73 | 22 | 5 | 32 | 16.00% | 10.78% |
| 184 | ATAD5 | 29161503 | A>G | 11080134 | GLU_GLY | 135 | 45 | 40 | 15 | 70 | 35.00% | 34.22% |
| 185 | ATAD5 | 29161845 | G>A | 17826219 | ARG_LYS | 249 | 73 | 22 | 5 | 32 | 16.00% | 10.76% |
| 186 | ATAD5 | 29167653 | A>C | 3764421 | ASN_HIS | 699 | 73 | 22 | 5 | 32 | 16.00% | 10.80% |
| 187 | ATAD5 | 29214387 | T>C | 11657270 | TYR_HIS | 1419 | 73 | 22 | 5 | 32 | 16.00% | 10.73% |
| 188 | TEFM | 29226228 | T>C | 2433 | ILE_VAL | 348 | 73 | 22 | 5 | 32 | 16.00% | 10.79% |
| 189 | NF1_OMG | 29623288 | C>T | 11080149 | GLY_ASP | 21 | 69 | 29 | 2 | 33 | 16.50% | 12.94% |
| 190 | COPRS | 30183857 | T>C | 8068049 | SER_GLY | 43 | 1 | 27 | 72 | 171 | 85.50% | 87.82% |
| 191 | UTP6 | 30222002 | T>C | 3760454 | GLN_ARG | 69 | 25 | 48 | 27 | 102 | 51.00% | 46.46% |
| 192 | LRRC37B | 30372739 | C>T | 1638299 | ALA_VAL | 715 | 35 | 15 | 0 | 15 | 15.00% | 11.52% |
| 193 | RHBDL3 | 30625205 | G>A | 4795690 | VAL_MET | 255 | 68 | 29 | 3 | 35 | 17.50% | 15.50% |
| 194 | ZNF207 | 30692396 | G>T | 3795244 | ALA_SER | 240 | 81 | 19 | 0 | 19 | 9.50% | 6.28% |
| 195 | CCL11 | 32612894 | G>A | 1129844 | ALA_THR | 23 | 65 | 29 | 6 | 41 | 20.50% | 18.32% |
| 196 | CCL8 | 32647831 | A>C | 1133763 | LYS_GLN | 69 | 76 | 22 | 2 | 26 | 13.00% | 15.51% |
| 197 | C17orf102 | 32904586 | C>T | 887230 | ARG_LYS | 155 | 3 | 29 | 68 | 165 | 82.50% | 84.52% |
| 198 | C17orf102 | 32906008 | C>G | 58529418 | GLY_ARG | 98 | 87 | 12 | 1 | 14 | 7.00% | 7.82% |
| 199 | C17orf102 | 32906038 | G>C | 117721431 | ARG_GLY | 88 | 87 | 13 | 0 | 13 | 6.50% | 6.50% |
| 200 | CCT6B | 33269648 | C>G | 2230553 | GLY_ALA | 210 | 50 | 43 | 7 | 57 | 28.50% | 36.98% |
| 201 | CCT6B | 33286664 | A>G | 2230552 | VAL_ALA | 48 | 60 | 33 | 7 | 47 | 23.50% | 20.52% |
| 202 | CCT6B | 33288363 | C>T | 9635769 | ARG_GLN | 17 | 23 | 50 | 27 | 104 | 52.00% | 57.89% |
| 203 | CCT6B_ZNF830 | 33288882 | T>G | 931196 | HIS_GLN | 99 | 0 | 4 | 96 | 196 | 98.00% | 98.09% |
| 204 | CCT6B_ZNF830 | 33289046 | G>C | 3744355 | SER_THR | 154 | 77 | 22 | 1 | 24 | 12.00% | 8.81% |
| 205 | RAD51D_RAD51L3-RFFL | 33433487 | C>T | 4796033 | ARG_GLN | 185 | 70 | 27 | 3 | 33 | 16.50% | 16.06% |
| 206 | FNDC8 | 33448818 | T>C | 1871892 | SER_PRO | 36 | 4 | 41 | 55 | 151 | 75.50% | 67.92% |
| 207 | NLE1 | 33464864 | C>T | 7215209 | ARG_LYS | 169 | 2 | 38 | 60 | 158 | 79.00% | 71.70% |
| 208 | NLE1 | 33469279 | G>C | 1471615 | PRO_ALA | 6 | 0 | 2 | 80 | 162 | 98.78% | 97.99% |
| 209 | UNC45B | 33477242 | G>A | 80100968 | LYS | 127 | 83 | 16 | 1 | 18 | 9.00% | 9.45% |
| 210 | UNC45B | 33513337 | T>A | 11654824 | ILE_ASN | 850 | 84 | 16 | 0 | 16 | 8.00% | 7.11% |
| 211 | SLFN5 | 33592591 | C>T | 11651240 | PRO_LEU | 787 | 30 | 59 | 11 | 81 | 40.50% | 46.89% |
| 212 | SLFN5 | 33592621 | C>T | 2291189 | ALA_VAL | 797 | 82 | 17 | 1 | 19 | 9.50% | 5.32% |
| 213 | SLFN11 | 33689926 | T>C | 4796077 | ASN_ASP | 301 | 0 | 0 | 100 | 200 | 100.00% | 96.65% |
| 214 | SLFN11 | 33690466 | C>A | 12453150 | VAL_PHE | 121 | 25 | 44 | 31 | 106 | 53.00% | 53.84% |
| 215 | SLFN11 | 33690619 | G>T | 72825958 | PRO_THR | 70 | 69 | 26 | 5 | 36 | 18.00% | 17.59% |
| 216 | SLFN12 | 33749546 | A>G | 2586514 | CYS_ARG | 168 | 13 | 41 | 46 | 133 | 66.50% | 59.99% |
| 217 | SLFN12 | 33749919 | A>C | 1849733 | SER_ARG | 43 | 24 | 47 | 29 | 105 | 52.50% | 49.10% |
| 218 | SLFN13 | 33768354 | C>T | 3744371 | GLU_LYS | 652 | 22 | 46 | 32 | 110 | 55.00% | 52.67% |
| 219 | SLFN13 | 33768383 | T>A | 11657183 | ASP_VAL | 642 | 72 | 26 | 2 | 30 | 15.00% | 11.86% |
| 220 | SLFN13 | 33768937 | C>T | 114866073 | ALA_THR | 523 | 66 | 29 | 2 | 33 | 17.01% | 19.65% |
| 221 | SLFN13 | 33769038 | C>A | 62078111 | ARG_LEU | 489 | 86 | 14 | 0 | 14 | 7.00% | 15.19% |
| 222 | SLFN13 | 33770802 | C>T | 10512469 | none |  | 71 | 27 | 2 | 31 | 15.50% | 19.23% |
| 223 | SLFN13 | 33772658 | G>T | 8072510 | TYR_stop | 14 | 81 | 18 | 1 | 20 | 10.00% | 10.49% |
| 224 | SLFN13 | 33772689 | T>C | 12943866 | ASN_SER | 4 | 76 | 23 | 1 | 25 | 12.50% | 10.00% |
| 225 | SLFN12L | 33802156 | T>G | 3744372 | TYR_SER | 518 | 73 | 23 | 4 | 31 | 15.50% | 14.91% |
| 226 | SLFN12L | 33805150 | T>C | 2304968 | TYR_CYS | 383 | 9 | 39 | 52 | 143 | 71.50% | 69.11% |
| 227 | SLFN12L | 33805180 | G>C | 2304967 | ALA_GLY | 373 | 73 | 23 | 4 | 31 | 15.50% | 14.58% |
| 228 | SLFN14 | 33875262 | T>A | 8073060 | TYR_PHE | 912 | 23 | 22 | 6 | 34 | 33.33% | 33.07% |
| 229 | SLFN14 | 33881631 | T>C | 321612 | LYS_GLU | 385 | 9 | 24 | 18 | 60 | 58.82% | 59.66% |
| 230 | SLFN14 | 33881718 | G>A | 321613 | PRO_SER | 356 | 9 | 24 | 18 | 60 | 58.82% | 59.80% |
| 231 | SLFN14 | 33884804 | T>C | 10512472 | GLN_ARG | 93 | 35 | 15 | 2 | 19 | 18.27% | 18.50% |
| 232 | GAS2L2 | 34072555 | G>A | 3744374 | ALA_VAL | 654 | 56 | 36 | 8 | 52 | 26.00% | 23.85% |
| 233 | GAS2L2 | 34077232 | G>A | 11654604 | ALA_VAL | 164 | 65 | 31 | 4 | 39 | 19.50% | 14.91% |
| 234 | MMP28_C17orf50 | 34091078 | C>G | 4795087 | ASP_GLU | 22 | 31 | 20 | 3 | 26 | 24.07% | 19.80% |
| 235 | C17orf66 | 34182341 | G>A | 2306630 | SER_PHE | 480 | 63 | 32 | 5 | 42 | 21.00% | 12.89% |
| 236 | RDM1 | 34252537 | A>C | 2251660 | CYS_TRP | 127 | 63 | 30 | 7 | 44 | 22.00% | 14.70% |
| 237 | LYZL6 | 34261831 | A>G | 9754 | PHE_SER | 139 | 64 | 32 | 4 | 40 | 20.00% | 14.67% |
| 238 | CCL15_CCL15-CCL14 | 34328461 | A>G | 854625 | ILE_THR | 24 | 1 | 11 | 88 | 187 | 93.50% | 95.89% |
| 239 | CCL23 | 34340284 | C>T | 1003645 | VAL_MET | 123 | 4 | 26 | 70 | 166 | 83.00% | 81.03% |
| 240 | CCL4 | 34432664 | T>A | 1719152 | SER_THR | 80 | 53 | 41 | 6 | 53 | 26.50% | 23.22% |
| 241 | TBC1D3B | 34499247 | T>C | 199912858 | LYS_ARG | 155 | 79 | 18 | 0 | 18 | 9.28% | 1.79% |
| 242 | TBC1D3G | 34797608 | T>C | 368112572 | SER_GLY | 510 | 40 | 13 | 0 | 13 | 12.26% | 34.45% |
| 243 | ZNHIT3_MYO19 | 34854280 | G>A | 2306590 | LEU_PHE | 863 | 30 | 56 | 14 | 84 | 42.00% | 41.38% |
| 244 | MYO19 | 34871721 | T>C | 2306595 | ASN_SER | 176 | 87 | 13 | 0 | 13 | 6.50% | 5.13% |
| 245 | PIGW | 34893326 | A>G | 72818370 | ASN_ASP | 126 | 87 | 13 | 0 | 13 | 6.50% | 5.23% |
| 246 | MRM1 | 34958598 | G>C | 78943308 | CYS_SER | 120 | 87 | 13 | 0 | 13 | 6.50% | 4.89% |
| 247 | ACACA_C17orf78 | 35743010 | C>G | 1714987 | THR_SER | 152 | 66 | 27 | 7 | 41 | 20.50% | 21.29% |
| 248 | TADA2A | 35771468 | C>T | 7211875 | PRO_SER | 6 | 2 | 28 | 70 | 168 | 84.00% | 85.96% |
| 249 | SYNRG | 35937637 | T>C | 12602536 | THR_ALA | 222 | 65 | 33 | 2 | 37 | 18.50% | 17.67% |
| 250 | SYNRG | 35956391 | G>C | 12944821 | ALA_GLY | 40 | 69 | 29 | 2 | 33 | 16.50% | 17.59% |
| 251 | DDX52 | 35984410 | T>C | 7216445 | MET_VAL | 403 | 3 | 37 | 60 | 157 | 78.50% | 79.76% |
| 252 | DDX52 | 36003359 | A>G | 3813910 | none |  | 5 | 36 | 59 | 154 | 77.00% | 78.85% |
| 253 | MRPL45 | 36453208 | T>C | 149988943 | PHE_SER | 20 | 69 | 29 | 2 | 33 | 16.50% | 13.65% |
| 254 | MRPL45 | 36478388 | G>T | 73302948 | none |  | 82 | 18 | 0 | 18 | 9.00% | 8.47% |
| 255 | MRPL45 | 36478450 | G>T | 34749623 | GLY_VAL | 248 | 89 | 10 | 1 | 12 | 6.00% | 5.46% |
| 256 | GPR179 | 36486004 | T>C | 55727040 | LYS_GLU | 1150 | 89 | 9 | 2 | 13 | 6.50% | 5.46% |
| 257 | GPR179 | 36486802 | G>A | 72832277 | ARG_TRP | 884 | 89 | 10 | 1 | 12 | 6.00% | 6.48% |
| 258 | ARHGAP23 | 36638388 | A>G | 11867891 | none |  | 30 | 23 | 3 | 29 | 25.89% | 33.79% |
| 259 | C17orf96 | 36830459 | G>A | 111565436 | PRO_LEU | 97 | 33 | 15 | 3 | 21 | 20.59% | 33.04% |
| 260 | C17orf96 | 36830562 | G>C | 79676758 | LEU_VAL | 63 | 35 | 11 | 4 | 19 | 19.00% | 20.73% |
| 261 | CISD3 | 36889559 | C>T | 2879097 | ARG_CYS | 79 | 35 | 13 | 5 | 23 | 21.70% | 22.92% |
| 262 | PSMB3 | 36909499 | A>T | 4907 | MET_LEU | 34 | 83 | 14 | 3 | 20 | 10.00% | 9.29% |
| 263 | C17orf98 | 36997461 | G>A | 7210156 | PRO_LEU | 61 | 86 | 14 | 0 | 14 | 7.00% | 7.27% |
| 264 | LASP1 | 37054772 | C>T | 1130638 | ASN | 63 | 17 | 23 | 10 | 43 | 43.00% | 44.48% |
| 265 | FBXO47 | 37101380 | T>C | 9906595 | GLN_ARG | 209 | 0 | 13 | 87 | 187 | 93.50% | 92.76% |
| 266 | PLXDC1_LOC100131347 | 37224211 | C>T | 75117355 | ARG_HIS | 462 | 86 | 14 | 0 | 14 | 7.00% | 8.01% |
| 267 | ARL5C | 37316988 | T>C | 16522 | GLN_ARG | 116 | 82 | 18 | 0 | 18 | 9.00% | 14.83% |
| 268 | ARL5C | 37319029 | T>G | 544198 | MET_LEU | 64 | 68 | 32 | 0 | 32 | 16.00% | 20.68% |
| 269 | ARL5C | 37319065 | C>T | 657672 | VAL_MET | 52 | 82 | 18 | 0 | 18 | 9.00% | 14.74% |
| 270 | ARL5C | 37319103 | T>C | 657723 | ASN_SER | 39 | 67 | 32 | 0 | 32 | 16.16% | 21.90% |
| 271 | STARD3 | 37813338 | T>C | 2941515 | PHE | 99 | 88 | 11 | 1 | 13 | 6.50% | 5.30% |
| 272 | STARD3 | 37814080 | G>A | 1877031 | ARG_GLN | 117 | 22 | 36 | 42 | 120 | 60.00% | 67.02% |
| 273 | STARD3 | 37816461 | T>C | 2952149 | none |  | 88 | 11 | 1 | 13 | 6.50% | 5.30% |
| 274 | ERBB2 | 37879588 | A>G | 1136201 | ILE_VAL | 625 | 56 | 37 | 7 | 51 | 25.50% | 23.96% |
| 275 | ERBB2 | 37884037 | C>G | 1058808 | PRO_ALA | 1140 | 19 | 40 | 41 | 122 | 61.00% | 67.33% |
| 276 | GRB7 | 37898543 | T>C | 2952142 | CYS_ARG | 20 | 0 | 0 | 100 | 200 | 100.00% | 99.99% |
| 277 | ZPBP2 | 38028634 | G>T | 11557467 | SER_ILE | 151 | 32 | 44 | 24 | 92 | 46.00% | 50.08% |
| 278 | GSDMB | 38062196 | G>A | 2305480 | PRO_SER | 298 | 33 | 45 | 22 | 89 | 44.50% | 45.31% |
| 279 | GSDMB | 38062217 | C>T | 2305479 | GLY_ARG | 291 | 33 | 43 | 24 | 91 | 45.50% | 49.31% |
| 280 | GSDMB | 38064469 | T>C | 11078928 | none |  | 33 | 45 | 22 | 89 | 44.50% | 44.88% |
| 281 | GSDMA | 38121993 | G>A | 3894194 | ARG_GLN | 18 | 35 | 38 | 27 | 92 | 46.00% | 47.31% |
| 282 | GSDMA | 38122680 | G>T | 7212938 | VAL_LEU | 128 | 29 | 42 | 29 | 100 | 50.00% | 51.29% |
| 283 | GSDMA | 38122686 | G>A | 7212944 | GLU_LYS | 130 | 49 | 36 | 15 | 66 | 33.00% | 33.85% |
| 284 | GSDMA | 38131187 | C>A | 56030650 | THR_ASN | 314 | 39 | 38 | 23 | 84 | 42.00% | 46.68% |
| 285 | CDC6 | 38457151 | G>A | 13706 | VAL_ILE | 441 | 71 | 29 | 0 | 29 | 14.50% | 10.84% |
| 286 | TNS4 | 38640744 | C>T | 2290207 | SER_ASN | 498 | 48 | 42 | 10 | 62 | 31.00% | 25.25% |
| 287 | TNS4 | 38645125 | A>G | 3764424 | LEU_PRO | 179 | 48 | 43 | 9 | 61 | 30.50% | 25.79% |
| 288 | CCR7 | 38715186 | T>C | 2228015 | MET_VAL | 7 | 89 | 11 | 0 | 11 | 5.50% | 3.99% |
| 289 | KRT24 | 38855772 | T>C | 2462961 | LYS_GLU | 429 | 30 | 46 | 24 | 94 | 47.00% | 44.94% |
| 290 | KRT24 | 38857446 | C>A | 874889 | MET_ILE | 267 | 30 | 48 | 22 | 92 | 46.00% | 43.84% |
| 291 | KRT25 | 38911327 | C>G | 35076248 | GLY_ALA | 66 | 74 | 25 | 1 | 27 | 13.50% | 11.30% |
| 292 | KRT25 | 38911363 | G>A | 12951399 | SER_LEU | 54 | 44 | 44 | 12 | 68 | 34.00% | 41.45% |
| 293 | KRT26 | 38926002 | G>A | 16966256 | none |  | 71 | 28 | 1 | 30 | 15.00% | 12.03% |
| 294 | KRT26 | 38928014 | A>G | 9898164 | TRP_ARG | 118 | 74 | 25 | 1 | 27 | 13.50% | 11.30% |
| 295 | KRT27 | 38935812 | A>G | 981684 | ILE_THR | 305 | 21 | 55 | 24 | 103 | 51.50% | 54.44% |
| 296 | KRT27 | 38935876 | C>T | 17558532 | ALA_THR | 284 | 62 | 35 | 3 | 41 | 20.50% | 17.25% |
| 297 | KRT27 | 38936659 | C>T | 17558560 | GLY_SER | 193 | 41 | 46 | 13 | 72 | 36.00% | 43.60% |
| 298 | KRT27 | 38938316 | C>T | 12453124 | GLU_LYS | 144 | 73 | 26 | 1 | 28 | 14.00% | 11.37% |
| 299 | KRT27 | 38938591 | C>G | 2469826 | SER_THR | 52 | 53 | 38 | 9 | 56 | 28.00% | 25.84% |
| 300 | KRT28 | 38955961 | G>A | 4624233 | ALA_VAL | 62 | 62 | 33 | 5 | 43 | 21.50% | 19.01% |
| 301 | KRT28 | 38955991 | C>T | 7209228 | GLY_ASP | 52 | 75 | 24 | 1 | 26 | 13.00% | 17.66% |
| 302 | KRT10_TMEM99 | 38978462 | C>T | 77919366 | GLY_SER | 126 | 51 | 43 | 6 | 55 | 27.50% | 23.38% |
| 303 | KRT10_TMEM99 | 38978536 | A>C | 4261597 | ILE_SER | 101 | 0 | 0 | 100 | 200 | 100.00% | 99.85% |
| 304 | TMEM99 | 38991003 | T>C | 10558 | TYR_HIS | 79 | 65 | 31 | 4 | 39 | 19.50% | 17.08% |
| 305 | TMEM99 | 38991052 | T>G | 1044806 | LEU_ARG | 95 | 71 | 27 | 2 | 31 | 15.50% | 19.62% |
| 306 | KRT12 | 39023381 | G>A | 17566772 | ARG_TRP | 20 | 85 | 15 | 0 | 15 | 7.50% | 8.37% |
| 307 | KRT12 | 39023396 | G>A | 11650915 | PRO_SER | 15 | 45 | 43 | 12 | 67 | 33.50% | 34.99% |
| 308 | KRT20 | 39041052 | C>T | 7212483 | SER_ASN | 129 | 84 | 16 | 0 | 16 | 8.00% | 8.50% |
| 309 | KRT23 | 39084504 | T>C | 9257 | THR_ALA | 303 | 28 | 48 | 24 | 96 | 48.00% | 40.09% |
| 310 | KRT39 | 39114962 | C>T | 7213256 | ARG_GLN | 456 | 73 | 25 | 2 | 29 | 14.50% | 15.90% |
| 311 | KRT39 | 39116728 | G>A | 17843021 | THR_MET | 341 | 79 | 20 | 1 | 22 | 11.00% | 12.87% |
| 312 | KRT40 | 39134528 | G>A | 16968862 | SER_LEU | 406 | 78 | 20 | 2 | 24 | 12.00% | 12.76% |
| 313 | KRT40 | 39135084 | A>G | 8064733 | TRP_ARG | 390 | 56 | 39 | 5 | 49 | 24.50% | 27.44% |
| 314 | KRT40 | 39135089 | G>A | 11649834 | THR_MET | 388 | 77 | 21 | 2 | 25 | 12.50% | 13.61% |
| 315 | KRT40 | 39135207 | A>G | 150812789 | CYS_ARG | 349 | 55 | 40 | 5 | 50 | 25.00% | 23.29% |
| 316 | KRT40 | 39137104 | A>G | 9908389 | MET_THR | 303 | 56 | 39 | 5 | 49 | 24.50% | 27.31% |
| 317 | KRT40 | 39137154 | C>G | 721958 | GLU_ASP | 286 | 56 | 39 | 5 | 49 | 24.50% | 27.31% |
| 318 | KRT40 | 39137297 | C>T | 721957 | CYS_TYR | 265 | 22 | 55 | 23 | 101 | 50.50% | 52.53% |
| 319 | KRT40 | 39137387 | C>T | 2010027 | ARG_HIS | 235 | 56 | 39 | 5 | 49 | 24.50% | 27.31% |
| 320 | KRT40 | 39139370 | G>A | 9908304 | THR_MET | 153 | 56 | 39 | 5 | 49 | 24.50% | 27.32% |
| 321 | KRT40 | 39140221 | C>T | 1510068 | SER_ASN | 102 | 56 | 39 | 5 | 49 | 24.50% | 27.31% |
| 322 | KRT40 | 39140272 | A>G | 17843015 | PHE_SER | 85 | 85 | 15 | 0 | 15 | 7.50% | 10.62% |
| 323 | KRT40 | 39140417 | T>C | 1510069 | THR_ALA | 37 | 56 | 39 | 5 | 49 | 24.50% | 27.35% |
| 324 | KRTAP3-2 | 39155969 | A>G | 3813050 | ILE_THR | 46 | 86 | 14 | 0 | 14 | 7.00% | 8.86% |
| 325 | KRTAP3-2 | 39156027 | G>A | 3829598 | ARG_CYS | 27 | 66 | 30 | 4 | 38 | 19.00% | 17.46% |
| 326 | KRTAP3-2 | 39156084 | T>C | 9897046 | SER_GLY | 8 | 86 | 14 | 0 | 14 | 7.00% | 8.97% |
| 327 | KRTAP1-3 | 39190758 | T>C | 62622847 | ILE_VAL | 106 | 71 | 24 | 4 | 32 | 16.16% | 16.22% |
| 328 | KRTAP1-1 | 39197499 | G>A | 3213755 | GLN_stop | 51 | 70 | 27 | 3 | 33 | 16.50% | 15.73% |
| 329 | KRTAP2-3 | 39216019 | G>A | 12937861 | ALA_VAL | 95 | 67 | 13 | 2 | 17 | 10.37% | 12.99% |
| 330 | KRTAP4-7 | 39240504 | A>G | 11655310 | SER_GLY | 16 | 12 | 49 | 39 | 127 | 63.50% | 62.08% |
| 331 | KRTAP4-7 | 39240511 | A>T | 383835 | ASP_VAL | 18 | 13 | 56 | 31 | 118 | 59.00% | 60.88% |
| 332 | KRTAP4-7 | 39240661 | C>G | 11650484 | THR_SER | 68 | 12 | 49 | 39 | 127 | 63.50% | 63.40% |
| 333 | KRTAP4-7 | 39240790 | G>A | 11650261 | ARG_HIS | 111 | 61 | 31 | 0 | 31 | 16.85% | 13.98% |
| 334 | KRTAP4-8 | 39253835 | C>T | 72625995 | ALA_THR | 168 | 15 | 41 | 34 | 109 | 60.56% | 56.15% |
| 335 | KRTAP4-8 | 39253886 | G>C | 78550130 | LEU_VAL | 151 | 13 | 44 | 29 | 102 | 59.30% | 63.78% |
| 336 | KRTAP4-8 | 39253960 | C>T | 144672535 | ARG_HIS | 126 | 18 | 36 | 23 | 82 | 53.25% | 4.13% |
| 337 | KRTAP4-8 | 39253969 | T>G | 147906218 | ASN_THR | 123 | 15 | 41 | 25 | 91 | 56.17% | 1.65% |
| 338 | KRTAP4-8 | 39254149 | G>C | 201246375 | THR_SER | 63 | 87 | 11 | 0 | 11 | 5.61% | 1.03% |
| 339 | KRTAP4-8 | 39254247 | A>T | 137943557 | CYS_stop | 30 | 87 | 13 | 0 | 13 | 6.50% | 4.96% |
| 340 | KRTAP4-8 | 39254257 | G>T | 142487897 | PRO_HIS | 27 | 87 | 13 | 0 | 13 | 6.50% | 4.96% |
| 341 | KRTAP4-9 | 39261693 | A>T | 113059833 | ASP_VAL | 18 | 88 | 12 | 0 | 12 | 6.00% | 20.54% |
| 342 | KRTAP4-9 | 39261933 | C>T | 7207685 | ALA_VAL | 98 | 7 | 45 | 46 | 137 | 69.90% | 71.39% |
| 343 | KRTAP4-11 | 39274069 | G>C | 349771 | ARG_GLY | 167 | 9 | 52 | 38 | 128 | 64.65% | 62.20% |
| 344 | KRTAP4-11 | 39274364 | T>G | 425784 | ARG_SER | 68 | 83 | 17 | 0 | 17 | 8.50% | 11.04% |
| 345 | KRTAP4-11 | 39274518 | C>T | 9897031 | ARG_GLN | 17 | 6 | 43 | 51 | 145 | 72.50% | 72.73% |
| 346 | KRTAP4-6 | 39296254 | C>T | 72483263 | none |  | 11 | 87 | 1 | 89 | 44.95% | 45.03% |
| 347 | KRTAP4-6 | 39296361 | A>G | 146882220 | SER_PRO | 127 | 19 | 80 | 0 | 80 | 40.40% | 43.59% |
| 348 | KRTAP4-6 | 39296412 | T>A | 200470462 | SER_CYS | 110 | 76 | 23 | 0 | 23 | 11.62% | 21.72% |
| 349 | KRTAP4-6 | 39296466 | G>A | 35985080 | PRO_SER | 92 | 88 | 12 | 0 | 12 | 6.00% | 6.97% |
| 350 | KRTAP4-5 | 39305646 | C>T | 1846044 | CYS_TYR | 125 | 86 | 13 | 0 | 13 | 6.57% | 7.42% |
| 351 | KRTAP4-5 | 39305785 | A>T | 411367 | CYS_SER | 79 | 30 | 70 | 0 | 70 | 35.00% | 1.49% |
| 352 | KRTAP4-5 | 39305820 | C>T | 238830 | ARG_HIS | 67 | 85 | 13 | 1 | 15 | 7.58% | 5.52% |
| 353 | KRTAP4-5 | 39305956 | G>A | 1497383 | ARG_CYS | 22 | 20 | 63 | 17 | 97 | 48.50% | 48.81% |
| 354 | KRTAP4-3 | 39324333 | T>A | 12953139 | GLN_LEU | 31 | 80 | 20 | 0 | 20 | 10.00% | 3.57% |
| 355 | KRTAP4-2 | 39334133 | T>C | 389784 | TYR_CYS | 95 | 1 | 14 | 85 | 184 | 92.00% | 92.69% |
| 356 | KRTAP4-1 | 39340707 | C>T | 398825 | ALA_THR | 115 | 1 | 14 | 85 | 184 | 92.00% | 92.68% |
| 357 | KRTAP4-1 | 39340910 | T>C | 2320231 | HIS_ARG | 66 | 1 | 14 | 85 | 184 | 92.00% | 93.34% |
| 358 | KRTAP9-1 | 39346139 | A>G | 61743546 | MET_VAL | 1 | 55 | 33 | 5 | 43 | 23.12% | 22.97% |
| 359 | KRTAP9-1 | 39346518 | T>C | 238824 | ILE_THR | 127 | 1 | 14 | 85 | 184 | 92.00% | 92.79% |
| 360 | KRTAP9-2 | 39383012 | C>T | 9903833 | PRO_SER | 36 | 7 | 84 | 9 | 102 | 51.00% | 57.65% |
| 361 | KRTAP9-2 | 39383073 | G>C | 9902235 | CYS_SER | 56 | 6 | 43 | 51 | 145 | 72.50% | 74.60% |
| 362 | KRTAP9-3 | 39388811 | C>A | 112082369 | GLN_LYS | 20 | 63 | 29 | 4 | 37 | 19.27% | 17.85% |
| 363 | KRTAP9-8 | 39394674 | A>G | 71383390 | ASN_SER | 124 | 10 | 38 | 43 | 124 | 68.13% | 73.68% |
| 364 | KRTAP9-4 | 39406343 | A>G | 148655704 | ASN_SER | 124 | 87 | 13 | 0 | 13 | 6.50% | 8.11% |
| 365 | KRTAP9-4 | 39406409 | C>A | 2191379 | SER_TYR | 146 | 6 | 45 | 49 | 143 | 71.50% | 73.39% |
| 366 | KRTAP9-4 | 39406427 | T>C | 139224734 | PHE_SER | 152 | 85 | 15 | 0 | 15 | 7.50% | 11.50% |
| 367 | KRTAP16-1 | 39464046 | G>C | 2074286 | ALA_GLY | 487 | 50 | 45 | 5 | 55 | 27.50% | 28.02% |
| 368 | KRTAP16-1 | 39464487 | G>C | 2074285 | PRO_ARG | 340 | 42 | 50 | 8 | 66 | 33.00% | 31.86% |
| 369 | KRTAP16-1 | 39464736 | C>G | 2074284 | SER_THR | 257 | 50 | 45 | 5 | 55 | 27.50% | 27.49% |
| 370 | KRTAP16-1 | 39464779 | T>C | 72828119 | SER_GLY | 243 | 84 | 15 | 1 | 17 | 8.50% | 7.78% |
| 371 | KRTAP16-1 | 39465094 | C>T | 12453338 | VAL_ILE | 138 | 63 | 36 | 1 | 38 | 19.00% | 19.89% |
| 372 | KRTAP17-1 | 39471778 | C>T | 78413710 | GLY_ASP | 42 | 71 | 22 | 0 | 22 | 11.83% | 19.99% |
| 373 | KRT33A | 39503163 | G>A | 12937519 | ALA_VAL | 270 | 51 | 44 | 5 | 54 | 27.00% | 26.23% |
| 374 | KRT33B | 39521248 | A>C | 71373411 | TYR_ASP | 294 | 85 | 14 | 1 | 16 | 8.00% | 8.30% |
| 375 | KRT33B | 39525750 | C>T | 12450621 | GLU_LYS | 85 | 88 | 12 | 0 | 12 | 6.00% | 5.95% |
| 376 | KRT34 | 39535388 | T>C | 2071599 | HIS_ARG | 348 | 76 | 23 | 1 | 25 | 12.50% | 10.47% |
| 377 | KRT34 | 39535859 | A>G | 2239710 | ILE_THR | 280 | 12 | 46 | 42 | 130 | 65.00% | 65.81% |
| 378 | KRT31 | 39553547 | G>A | 6503627 | ALA_VAL | 82 | 85 | 14 | 1 | 16 | 8.00% | 8.23% |
| 379 | KRT37 | 39577215 | G>A | 8071814 | THR_MET | 422 | 85 | 14 | 1 | 16 | 8.00% | 8.42% |
| 380 | KRT37 | 39578424 | G>T | 2071607 | ALA_ASP | 306 | 64 | 35 | 1 | 37 | 18.50% | 17.12% |
| 381 | KRT37 | 39579059 | G>A | 78158550 | GLN_stop | 235 | 64 | 35 | 1 | 37 | 18.50% | 16.92% |
| 382 | KRT37 | 39579112 | G>A | 16966811 | ALA_VAL | 217 | 64 | 35 | 1 | 37 | 18.50% | 17.13% |
| 383 | KRT37 | 39580559 | T>A | 9916475 | SER_CYS | 73 | 30 | 47 | 23 | 93 | 46.50% | 47.93% |
| 384 | KRT37 | 39580562 | T>C | 9916484 | THR_ALA | 72 | 30 | 47 | 23 | 93 | 46.50% | 47.93% |
| 385 | KRT37 | 39580660 | T>C | 9916724 | ASN_SER | 39 | 30 | 47 | 23 | 93 | 46.50% | 48.03% |
| 386 | KRT37 | 39580739 | C>A | 9910204 | GLY_CYS | 13 | 52 | 40 | 8 | 56 | 28.00% | 31.09% |
| 387 | KRT38 | 39593768 | A>G | 897416 | SER_PRO | 423 | 0 | 19 | 81 | 181 | 90.50% | 90.17% |
| 388 | KRT38 | 39595484 | G>A | 148768443 | GLN_stop | 235 | 83 | 17 | 0 | 17 | 8.50% | 2.81% |
| 389 | KRT32 | 39616430 | G>T | 2604953 | PRO_THR | 427 | 0 | 17 | 83 | 183 | 91.50% | 94.34% |
| 390 | KRT32 | 39619078 | G>A | 2857258 | none |  | 83 | 14 | 2 | 18 | 9.09% | 9.22% |
| 391 | KRT32 | 39619094 | T>C | 2604955 | ASN_SER | 402 | 13 | 48 | 39 | 126 | 63.00% | 64.45% |
| 392 | KRT32 | 39619115 | G>A | 2071563 | THR_MET | 395 | 41 | 46 | 13 | 72 | 36.00% | 38.29% |
| 393 | KRT32 | 39619186 | G>C | 2604956 | ASP_GLU | 371 | 84 | 14 | 2 | 18 | 9.00% | 9.22% |
| 394 | KRT32 | 39619193 | C>T | 11078993 | ARG_GLN | 369 | 83 | 17 | 0 | 17 | 8.50% | 4.99% |
| 395 | KRT32 | 39620565 | C>T | 72830046 | ARG_HIS | 280 | 52 | 37 | 11 | 59 | 29.50% | 30.17% |
| 396 | KRT32 | 39622068 | G>T | 2071561 | SER_TYR | 222 | 22 | 52 | 26 | 104 | 52.00% | 49.55% |
| 397 | KRT32 | 39623363 | T>C | 3744786 | GLN_ARG | 72 | 43 | 45 | 12 | 69 | 34.50% | 32.76% |
| 398 | KRT35 | 39633349 | G>C | 2071601 | PRO_ALA | 443 | 17 | 55 | 28 | 111 | 55.50% | 55.50% |
| 399 | KRT35 | 39633354 | C>T | 12451652 | CYS_TYR | 441 | 66 | 33 | 1 | 35 | 17.50% | 16.94% |
| 400 | KRT35 | 39637244 | A>G | 743686 | SER_PRO | 36 | 17 | 55 | 28 | 111 | 55.50% | 55.31% |
| 401 | KRT36 | 39643340 | T>G | 11657323 | ASN_THR | 357 | 63 | 34 | 3 | 40 | 20.00% | 22.54% |
| 402 | KRT36 | 39643646 | G>A | 2301354 | THR_MET | 315 | 19 | 52 | 29 | 110 | 55.00% | 55.61% |
| 403 | KRT13 | 39659183 | G>A | 4796698 | none |  | 2 | 14 | 84 | 182 | 91.00% | 94.58% |
| 404 | KRT13 | 39659194 | T>C | 4796697 | THR_ALA | 298 | 0 | 1 | 99 | 199 | 99.50% | 99.43% |
| 405 | KRT13 | 39659913 | G>A | 9891361 | ALA_VAL | 187 | 3 | 26 | 71 | 168 | 84.00% | 90.03% |
| 406 | KRT13 | 39661366 | G>C | 760134 | ALA_GLY | 146 | 84 | 14 | 2 | 18 | 9.00% | 5.00% |
| 407 | KRT15 | 39670912 | G>C | 897420 | ALA_GLY | 421 | 0 | 0 | 100 | 200 | 100.00% | 99.98% |
| 408 | KRT15 | 39671724 | T>C | 2305556 | LYS_ARG | 416 | 57 | 35 | 8 | 51 | 25.50% | 21.87% |
| 409 | KRT15 | 39674641 | T>C | 1050784 | THR_ALA | 147 | 12 | 44 | 44 | 132 | 66.00% | 64.12% |
| 410 | KRT19 | 39680796 | T>A | 1869720 | none |  | 0 | 0 | 100 | 200 | 100.00% | 99.99% |
| 411 | KRT19 | 39684321 | G>C | 4602 | ALA_GLY | 60 | 12 | 44 | 44 | 132 | 66.00% | 64.26% |
| 412 | KRT14 | 39742807 | C>T | 3826550 | ALA_THR | 94 | 37 | 50 | 13 | 76 | 38.00% | 42.72% |
| 413 | KRT14 | 39742899 | C>T | 6503640 | CYS_TYR | 63 | 0 | 0 | 93 | 186 | 100.00% | 99.87% |

^1^ Exome sequencing identified SNPs found in 10 or more FM patients and in the region of chromosome 17 from 17p13.3 to 17q25.3**,**

expanded from Arnold et al. that covered 17p11.2 to 17q11.2 to cover additional genes. Chemokine genes (CCL4, CCL8, CCL1, and

CCL23) are shaded in grey.
