# Supplementary material for: SNPs in inflammatory genes CCL11, CCL4 and MEFV in a fibromyalgia family study
Source: PLoS One. 2018 Jun 21;13(6):e0198625. doi: 10.1371/journal.pone.0198625 (PMC6013222; doi:10.1371/journal.pone.0198625)
Supplement: S2 Table — (DOCX) [file pone.0198625.s002.docx]

**S2 Table. Transmission analysis of *CCL11* variant -146 C>T in 120 FM trios ^1^.**

| **SNP** | **Transmitted** | **Not Transmitted** | **P value** |
| --- | --- | --- | --- |
| C>T | 17 | 15 | NS |

^1^ rs111568837 was analyzed in 120 FM trios by TDT.
