# Supplementary material for: SNPs in inflammatory genes CCL11, CCL4 and MEFV in a fibromyalgia family study
Source: PLoS One. 2018 Jun 21;13(6):e0198625. doi: 10.1371/journal.pone.0198625 (PMC6013222; doi:10.1371/journal.pone.0198625)
Supplement: S3 Table — (DOCX) [file pone.0198625.s003.docx]

**S3 Table. *MEFV* SNPs identified in 220 fibromyalgia probands^1^.**

| **SNP ID** | **Variant** | **Exon** | **Phenotype** |
| --- | --- | --- | --- |
| rs11466018 | L110P | 2 | unknown |
| rs3743930 | E148Q | 2 | FMF, FMF atypical |
| rs224222 | R202Q | 2 | unknown |
| rs104895132 | A289V | 2 | FMF |
| rs104895112 | R329H | 3 | unknown |
| rs114466023 | P369S | 3 | unknown |
| rs11466024 | R408Q | 3 | unknown |
| rs104895151 | A457V | 5 | unknown |
| n/a | E526Q | 5 | unknown |
| rs11466045 | I591T | 9 | FMF |
| rs104895094 | K695R | 10 | FMF |
| rs28940579 | V726A | 10 | FMF |
| rs51732874 | A744S | 10 | FMF |

1 Thirteen variants were identified, six of which are associated with Familial

Mediterranean Fever (FMF).
