# Supplementary figures and images for: SNPs in inflammatory genes CCL11, CCL4 and MEFV in a fibromyalgia family study
Source: PLoS One. 2018 Jun 21;13(6):e0198625. doi: 10.1371/journal.pone.0198625 (PMC6013222; doi:10.1371/journal.pone.0198625)

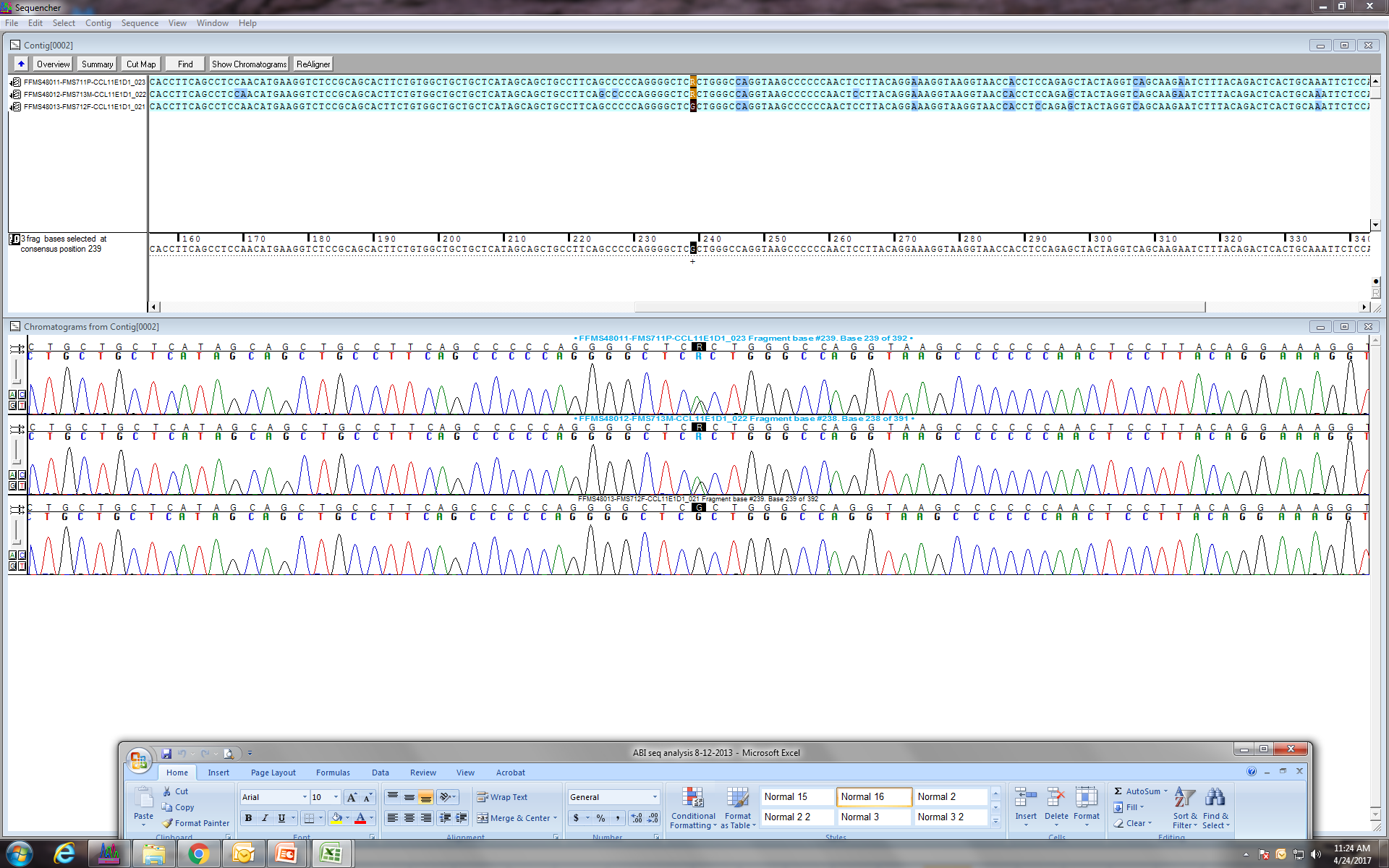


**S1 Fig. Example of sequence analysis of a FM patient heterozygous for rs1129844 on *CCL11*.**

Supplement: S1 Fig — (DOCX) [file pone.0198625.s004.docx]
