# Supplementary material for: SNPs in inflammatory genes CCL11, CCL4 and MEFV in a fibromyalgia family study
Source: PLoS One. 2018 Jun 21;13(6):e0198625. doi: 10.1371/journal.pone.0198625 (PMC6013222; doi:10.1371/journal.pone.0198625)
Supplement: S2 Fig — (DOCX) [file pone.0198625.s005.docx]

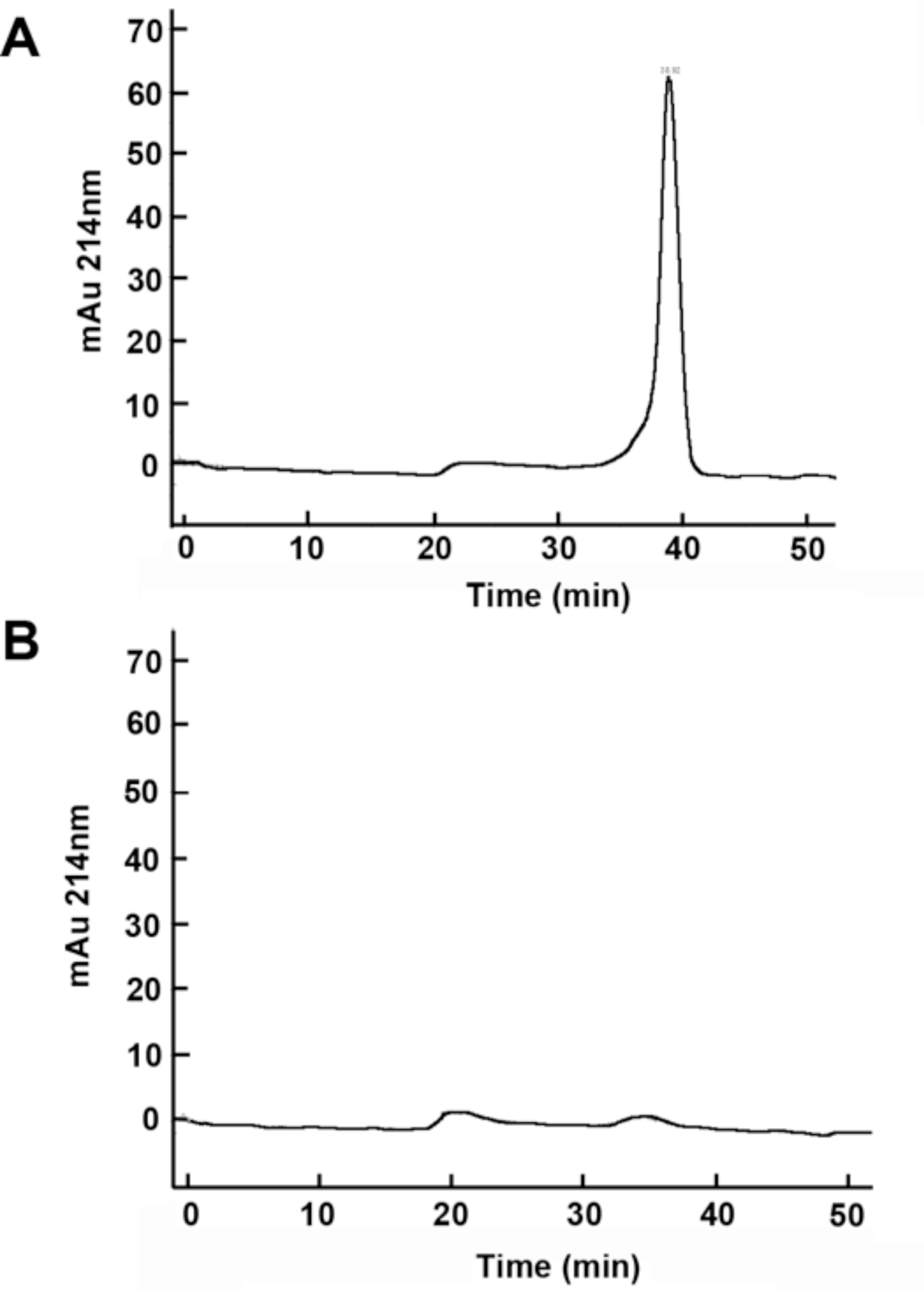


**S2 Fig.** **Protein size profiles of wild type CCL4 and varCCL4.** Size exclusion chromatography of WT CCL4 (**A**) and varCCL4 (**B**) on a Superdex 200 10/300 GL column in phosphate-buffered saline (PBS). Injected sample concentrations were 1.0 mg/mL (0.1 mL injected).
